# The Effectiveness of Noninvasive Biomarkers to Predict Hepatitis B-Related Significant Fibrosis and Cirrhosis: A Systematic Review and Meta-Analysis of Diagnostic Test Accuracy

**DOI:** 10.1371/journal.pone.0100182

**Published:** 2014-06-25

**Authors:** Xue-Ying Xu, Hong Kong, Rui-Xiang Song, Yu-Han Zhai, Xiao-Fei Wu, Wen-Si Ai, Hong-Bo Liu

**Affiliations:** 1 School of Public Health, China Medical University, Shenyang, PR China; 2 Department of Clinical Laboratory, Shengjing Hospital of China Medical University, Shenyang, PR China; 3 Department of Urinary Surgery, Changhai Hospital, Shanghai, PR China; Drexel University College of Medicine, United States of America

## Abstract

Noninvasive biomarkers have been developed to predict hepatitis B virus (HBV)-related fibrosis owing to the significant limitations of liver biopsy. Those biomarkers were initially derived from evaluation of hepatitis C virus (HCV)-related fibrosis, and their accuracy among HBV-infected patients was under constant debate. A systematic review was conducted on records in PubMed, EMBASE and the Cochrane Library electronic databases, up until April 1st, 2013, in order to systematically assess the effectiveness and accuracy of these biomarkers for predicting HBV-related fibrosis. The questionnaire for quality assessment of diagnostic accuracy studies (QUADAS) was used. Out of 115 articles evaluated for eligibility, 79 studies satisfied the pre-determined inclusion criteria for meta-analysis. Eventually, our final data set for the meta-analysis contained 30 studies. The areas under the SROC curve for APRI, FIB-4, and FibroTest of significant fibrosis were 0.77, 0.75, and 0.84, respectively. For cirrhosis, the areas under the SROC curve for APRI, FIB-4 and FibroTest were 0.75, 0.87, and 0.90, respectively. The heterogeneity of FIB-4 and FibroTest were not statistically significant. The heterogeneity of APRI for detecting significant fibrosis was affected by median age (*P* = 0.0211), and for cirrhosis was affected by etiology (*P* = 0.0159). Based on the analysis we claim that FibroTest has excellent diagnostic accuracy for identification of HBV-related significant fibrosis and cirrhosis. FIB-4 has modest benefits and may be suitable for wider scope implementation.

## Introduction

Chronic infection with hepatitis B virus (HBV) is an important global health problem. Approximately 350 million people are chronically infected with hepatitis B virus worldwide, especially in developing countries, 25% of whom will die from long term sequelae, such as cirrhosis, liver failure and hepatocellular carcinoma, resulting in 600,000 to one million deaths annually [1]. Patients who are suffering from significant hepatic inflammation and fibrosis are at high risk of those complications [2]. Assessment of liver significant fibrosis is critical to establishing effective clinical practice. It could be of great help for a doctor to determine patients' suitability and the optimal time for antiviral therapy to achieve the best curative effects as well as to prevent excessive medication [3]. In addition, early prediction of cirrhosis is beneficial to reducing complications in patients with chronic viral hepatitis [4].

Liver biopsy, an invasive technique, is the gold standard for the assessment of fibrosis. It has several disadvantages, such as patients' reluctance, pain, hemoperitoneum, and pneumothorax, etc. [5]. In addition, its accuracy in assessing fibrosis is questionable because of sampling errors and intra- and inter-observer variations [6]. Therefore, many people are beginning to realize the importance of prediction of liver fibrosis by noninvasive biomarkers.

Aspartate aminotransferase-to-platelet ratio index (APRI), the fibrosis index based on the 4 factors (FIB-4) and FibroTest are examples of noninvise biomarkers predicting liver fibrosis based on routinely available clinical parameters [7]. They were initially used in Western populations with hepatitis C virus (HCV) or HCV/ human immunodeficiency virus (HIV) co-infection [8] and had good performance. The area under the receiver operating characteristic (AUROC) curve of FibroTest for detecting significant fibrosis peaked out at 0.85 [9], and the AUROC curve of APRI and FIB-4 reached 0.80 [10] and 0.81 [11] respectively. For detecting cirrhosis, FibroTest also has the best result, and its AUROC curve topped out at 0.90 [12]. The AUROC curve of APRI and FIB-4 are 0.83 [13] and 0.89 [14], respectively. These three markers can be considered as “good”, even “better” markers, according to the criteria of Deeks JJ [15]. Consequently, the researchers were regularly conducting those markers to predict significant fibrosis and cirrhosis among HBV-infected patients. APRI was first used to predict significant fibrosis or cirrhosis in patients with HBeAg-negative chronic hepatitis B by Chrysanthos et al. [16]. They found APRI was strongly correlated to the fibrosis. Later FIB-4 and FibroTest were successively used to predict HBV-related fibrosis.

However, due to the fact that those markers were initially derived from evaluation of HCV-related fibrosis, their accuracy for HBV patients was under constant debate among the researchers. Some scholars indicated that all of those noninvasive markers were able to predict significant fibrosis or cirrhosis among HBV patients, and could potentially be used to decrease the number of liver biopsies [7]. Others maintained that those markers were not directly applicable to evaluation of HBV-related fibrosis because of the small AUROC curve [17]. Therefore, we decided to conduct this meta-analysis to assess the pooled performance of these biomarkers for prediction of significant fibrosis and cirrhosis among HBV-infected patients. It could provide the basis for future research and clinical application.

## Methods

### Literature Search

The review followed the Preferred Reporting Items for Systematic Reviews and Meta-Analyses (PRISMA) guidelines [18] (see Checklist S1 for PRISMA checklist). A protocol (see Text S1) was developed and systematic methods were used to identify relevant studies, assess study eligibility for inclusion, and evaluate study quality. Online database search was completed on PubMed, EMBASE and the Cochrane Library (01/2003-04/2013) for terms including the following: aspartate aminotransferase-to-platelet ratio index, APRI, fibrosis index based on the 4 factors, FIB-4, FibroTest, hepatitis B virus, HBV, Chronic hepatitis B, CHB, fibrosis and cirrhosis (see Text S2 for full search strategies). Additional studies were identified via a manual search for the referenced studies and review articles. EndNote X5 software was used to manage the references.

### Selection Criteria

Studies were included if they met the following inclusion criteria: (a) The study evaluated the performance of the APRI and/or FIB-4 and/or FibroTest for the prediction of fibrosis and/or cirrhosis in HBV infected patients. Studies on patients with other etiologies of liver disease were also included if data for HBV-infected patients could be independently extracted. In addition, special populations of HBV patients (*e.g*., HBV/HIV coinfection, HBV/HCV, and HBV/ hepatitis D virus [HDV]) were also included. (b) Liver biopsy was used to diagnose liver fibrosis as a golden standard. (c) Data could be extracted to construct at least one 2×2 table of test performance, based on some cutoff points of the APRI, FIB-4, and FibroTest for a fibrosis stage. (d) They assessed the diagnostic accuracy for fibrosis stage F≥2 or F≥4 according to METAVIR or a comparable staging system. (e) The study included at least 40 patients. Studies of smaller sample sizes were excluded due to concerns on their applicability.

### Data Extraction and Quality Assessment

Two reviewers (XYX and RXS) screened the downloaded titles and abstracts against the inclusion criteria. Two reviewers (XYX and HK) independently evaluated study eligibility, graded the study quality, and extracted data from the study. Any disagreements between the reviewers were resolved with detailed discussions between them together with a third reviewer (HBL). The parameters in our literature search included author, year of publication, region, method, patient gender, age, number of patients, underlying chronic liver disease etiology, histological scoring system, average length of liver specimen, time interval between biopsy and laboratory tests, prevalence of the fibrosis stage, as well as cutoff values to identify the fibrosis stage [13].

The quality of included studies was independently appraised by two reviewers (XYX and YHZ) using the quality assessment of diagnostic accuracy studies (QUADAS) questionnaire [19] (see Text S3). It could estimate the internal and external validity of diagnostic accuracy studies used in systematic reviews.

### Statistical Analysis and Data Synthesis

We extracted and tabulated the data in a series of 2×2 tables, which included sensitivity, specificity, positive predictive value (PPV) and negative predictive value (NPV) at each threshold value. The primary outcome was the identification of significant fibrosis, defined by METAVIR [20], Batts and Ludwig [21], and Scheuer [22] for stages F2 through F4, and Ishak [23] for stages F3 through F6. This gauge was chosen because significant fibrosis is often considered a threshold for the initiation of antiviral therapy [24]. We also assessed cirrhosis (METAVIR, Batts and Ludwig, and Scheuer F4, and Ishak F5-6). In order to provide clinically meaningful results, the metrics of diagnostic test accuracy were examined.

The SROC curve, generated using linear regression, represents the relationship between the true positive rate and false positive rate across these studies, albeit they may have used different test thresholds [25]. In this analysis, the area under SROC curve was examined according to Moses et al. [26], and each study was weighted with its sample size and with adjustment for the number of thresholds within each study [27].

The diagnostic odds ratio (DOR) describes the odds of a positive test in true disease cases compared with cases of no disease [15]. The summary DOR was calculated using a DerSimonian and Laird random-effects model on a logarithmic scale with a corresponding test of heterogeneity [28]. Because such analyses require a single measure of accuracy for each study and many studies reported multiple test thresholds, we calculate the average DOR among all thresholds for a given study [29]. We also calculated summary sensitivities and specificities using the bivariate meta-analytic approach [30]. Pairs of sensitivity and specificity for diagnostic thresholds are jointly analyzed, with any correlation that might exist between those two measures taken into account using a random-effects approach.

The heterogeneity (or the lack of homogeneity) of the results between studies was assessed statistically using the Cochran-Q and the quantity I2. I2 value describes the percentage of total variation across studies that is attributable to heterogeneity rather than chance [31]. A meta-regression was conducted to further explore the covariates that may induce heterogeneity, according to the following predefined characteristics: (a) study design (retrospective or prospective); (b) etiology (HBV, HBV[HBeAg negative], or co-infection with other virus); (c) length of liver specimen (≥10 mm, ≥15 mm, ≥20 mm, or not); (d) liver biopsy scoring system (METAVIR, Ishak, Scheuer, Batts and Ludwig, and the Chinese Hospital System); (e) QUADAS score; (f) sample size; (g) median age (≤30, 31–40,41–50, or >50); (h) percentage of males; (i) location of study (Europe or Asia); (j) prevalence of significant fibrosis/cirrhosis.

The potential publication bias was assessed using the Deeks funnel plots (the logarithm of the DOR plotted against 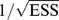) [32]. 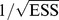
is proportional to the square root of (1/n1+1/n2), where n1 is the number diseased and n2 not diseased. Data analyses were performed using the Meta-Disc software (v. 1.4).

## Results

### Search Results

**Figure 1:**
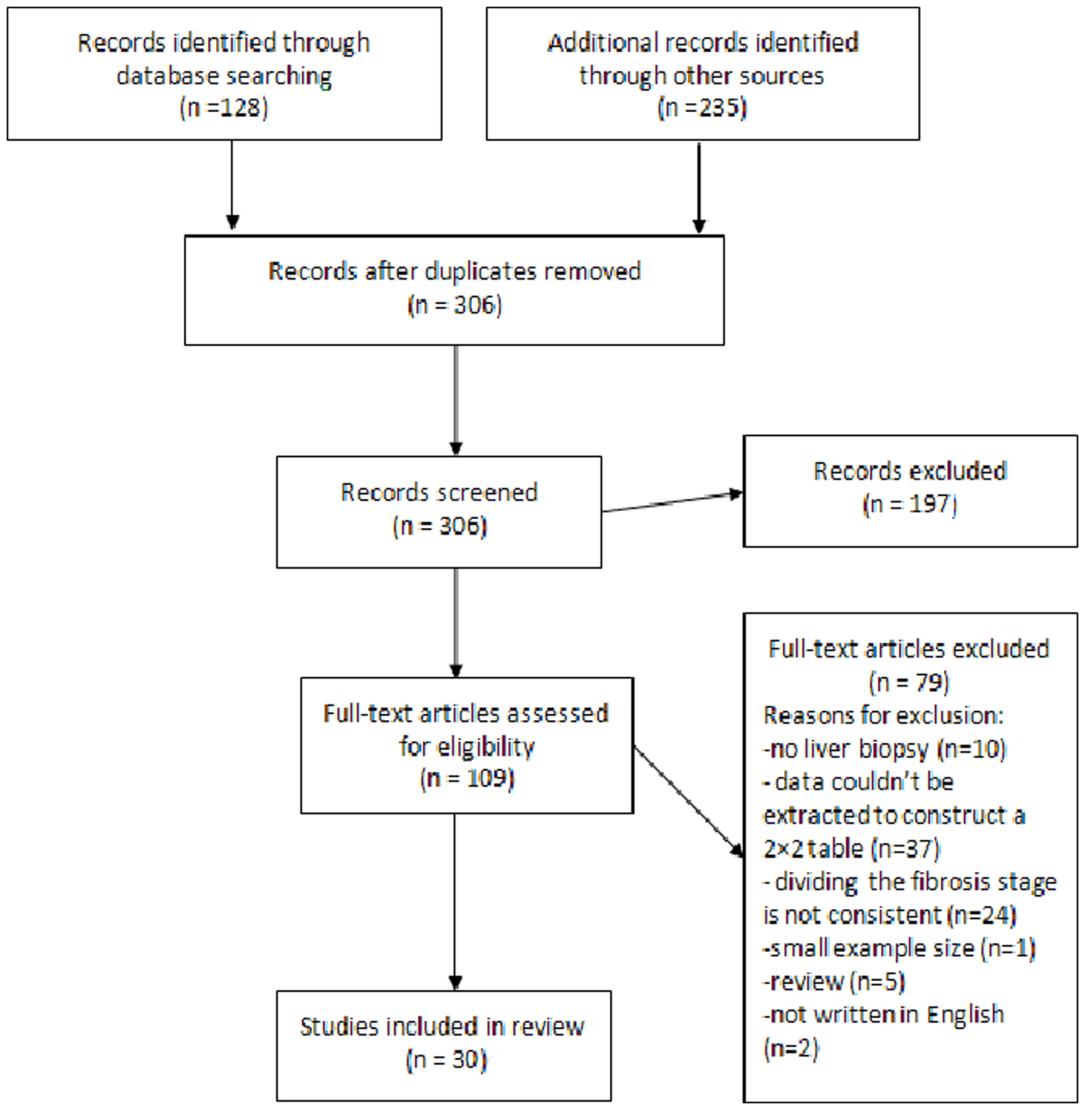
Flow diagram of article selection.

The study selection process is presented with a flow chart in Figure 1. 306 studies were retrieved with the described search strategies, of which 196 were excluded following title and abstract screening. The full texts of 110 potentially eligible reports were obtained for further assessment. Of those, 30 papers were included in the review following full-text screening (Table 1); 20 studies were related to the APRI [3], [16], [17], [33]-[49], 13 studies related to the FIB-4 [3], [7], [17], [39]–[46], [48], [50], and 11 studies related to the FibroTest [47]–[49], [51]–[58].

**Table 1:** Characteristics of the 31 Studies Included in the Meta-analysis.

Test	Author, Year, Regin	Study/Center Description	N	Interval Between Biopsy&Predictive index	Mean Age (%male)	Etiology	Liver biopsy System	length of liver specimen	Prevalence F2-4/F4	QUADAS Score
APRI	Zhou, 2010, China	Retrospective, 4 centers	146	Same time	35(84%)	HBV	Scheuer	>15 mm	58%,10%	13
APRI	Lin, 2008, TaiWan	Retrospective, one center	48	Unclear	56(83%)	HBV	METAVIR	Unclear	NA,38%	12
APRI	Shin, 2008, Korea	Retrospective, one center	264	Unclear	28(87%)	HBV	METAVIR	22 mm	53%,3%	13
APRI	Kim, 2007, Korea	Retrospective, 2 centers	346	Same time	34(90%)	HBV	Batts Ludwing	Unclear	75%,23%	13
APRI	Chrysanthos, 2005, Greece	Retrospective, one center	205	Same time	51(75%)	HBV(eAg-)	Ishak	≥15 mm	60%,27%	12
APRI	Liu, 2007, China	Retrospective, 2 centers	444	<1 week	30(71%)	HBV	Scheuer	>10 mm	29%,6%	14
APRI	Guzelbulut, 2011, Turkey	Retrospective, one center	250	Unclear	39(58%)	HBV	Ishak	Unclear	26%,16%	11
APRI	Lesmana, 2011, Indonesia	Retrospective, one center	117	Unclear	41(54%)	HBV	METAVIR	≥15 mm	62%,3%	13
FIB-4	Kim, 2009, Korea	Retrospective, one center	668	<2 days	39(66%)	HBV	Batts Ludwing	≥15 mm	79%,34%	12
FIB-4	Zhang, 2010, China	Retrospective, one center	212	Unclear	31(88%)	HBV	Scheuer	20 mm	76%,21%	11
FibroTest	Myers, 2003, France	Retrospective, one center	209	<6 months	39(70%)	HBV+HDV	METAVIR	Unclear	29%,9%	14
FibroTest	Miailhes, 2011, France	Prospective, 2 centers	59	Unclear	43(84%)	HBV+HIV	METAVIR	58%≥15 mm	61%,20%	14
FibroTest	Bottero, 2009, France	Cohort, multicenter	108	<6 months	42(90%)	HBV+HIV/HDV	METAVIR	17.0±7.3 mm	56%,15%	10
FibroTest	Kim, 2012, Korea	Retrospective, one center	194	Same time	47(61%)	HBV	Batts Ludwing	≥20 mm	85%,39%	14
FibroTest	Stibbe, 2011, The Netherlands	Retrospective, one center	48	<6 months	37(73%)	HBV	METAVIR	≥20 mm	46%,10%	12
FibroTest	Park, 2013, Korea	Retrospective, 3 centers	330	Same time	44(61%)	HBV	Batts Ludwing	≥20 mm	80%,24%	13
FibroTest	Gui, 2008, China	Retrospective, one center	100	Same time	35(78%)	HBV	Ishak	≥15 mm	39%,12%	14
FibroTest	Kim, 2012, Korea	Retrospective, one center	170	Same time	45(60%)	HBV	Batts Ludwing	≥20 mm	71%,28%	12
APRI/FIB-4	Seto, 2011, China	Prospective, one center	237	Same time	32(67%)	HBV	Ishak	15–20 mm	32%,2%	13
APRI/FIB-4	Liu, 2012, China	Retrospective, 2 centers	114	Same time	38(80%)	HBV	China hospital	15–20 mm	51%,11%	14
APRI/FIB-4	Zhu, 2011, China	Retrospective, one center	175	<7 days	37(78%)	HBV	METAVIR	>15 mm	45%,17%	14
APRI/FIB-4	Wu, 2010, China	Retrospective, one center	78	Unclear	33(85%)	HBV	METAVIR	18.2±3.4 mm	41%,12%	13
APRI/FIB-4	Wang, 2012, China	Retrospective, one center	231	Same time	34(68%)	HBV	Scheuer	≥15 mm	29%,7%	14
APRI/FIB-4	Ucar, 2013, Turkey	Retrospective, one center	73	Same time	45(64%)	HBV	METAVIR	Unclear	56%,11%	14
APRI/FIB-4	Gumusay, 2011, Turkek	Prospective, one center	58	Unclear	41(57%)	HBV	Ishak	≥20 mm	17%,NA	13
APRI/FIB-4	Basar, 2013, Turkey	Retrospective, one center	76	Same time	45(45%)	HBV	METAVIR	≥10 mm	67%,17%	14
APRI/FIB-4	Wang, 2012, China	Retrospective, multicenter	349	Same time	37(92%)	HBV(eAg-)	Scheuer	≥10 mm	60%,7%	13
APRI/FIB-4	Liu, 2011, china	Retrospective, one center	623	<1 week	32(55%)	HBV	METAVIR	≥10 mm	35%,6%	14
APRI/FT	Sebastiani, 2011, Italy	Retrospective, multicenter	253	Same time	44(73%)	HBV+HDV	METAVIR	Unclear	58%,8%	13
APRI/FT	Sebastiani, 2007, Italy	Retrospective, one center	110	Same time	43(73%)	HBV	METAVIR	≥15 mm	68%,20%	12
APRI/FIB-4/FibroTest	Bonnard, 2010, France	Prospective, one center	59	Same time	35(69%)	HBV	METAVIR	21±6 mm	70%,24%	14

APRI, aspartate aminotransferase-to-platelet ratio index; FIB-4, fibrosis index based on the 4 factors; HBV, hepatitis B virus; HCV, hepatitis C virus; HDV, hepatitis D virus; QUADAS, The quality assessment of diagnostic accuracy studies.

### Characteristics of the Included Studies

In the twenty APRI studies, a total of 4,208 patients (median age 36 yr, 72% male) were included. The overall prevalence of significant fibrosis and cirrhosis were 47% (ranged 17%–70%) and 11% (7%–27%), respectively. The liver biopsy scoring system used to classify the histology varied. 10 studies used a METAVIR score, 4 studies used an Ishak score, 4 studies used a Scheuer score, 1 study used a Batts and Ludwig score, and 1 study used the Chinese Hospital System. Nineteen of these studies (N = 3,955) included HBV-infected patients without comorbid conditions [3], [16], [17], [33]–[48]. The one remaining study included special populations of patients such as HBV/HDV-coinfected patients (N = 253) [59]. According to the QUADAS scale, eight studies met all 14 requirements of this scale, nine studies met 13, two studies met 12, and one study met 11.

A total of 2,953 patients (median age 36 yr, 70% male) were included in the thirteen studies on FIB-4. The overall prevalence of significant fibrosis and cirrhosis were 53% (ranged 17%–76%) and 15% (11%–34%), respectively. All those studies (N = 2,953) included HBV-infected patients without comorbid conditions. The liver biopsy scoring system used to classify the histology varied. Six studies used a METAVIR score, three studies used a Scheuer score, two studies used an Ishak score, one study used a Batts and Ludwig score, and one study used the Chinese Hospital System. Seven studies met all 14 requirements of the QUADAS scale, 4 studies met 13, 1 study met 12, and one study met 11.

There were 1,640 patients (median age 42 yr, 69% male) used to assess the performance of FibroTest in eleven studies. The overall prevalence of significant fibrosis and cirrhosis were 63% (ranged 39%–85%) and 20% (8%–39%), respectively. Seven of these studies (N = 1,011) included HBV-infected patients without comorbid conditions [47], [48], [54]–[58]. The four remaining studies included special populations with HBV/HDV-coinfected patients (N = 462) [49], [51], HBV/HIV-coinfected patients (N = 59) [52], and HBV/HDV/HIV-coinfected patients (N = 108) [53]. According to the Quality Assessment of Diagnostic Accuracy Studies scale, we can see that 5 studies met all 14 requirements of this scale, 2 study met 13, 3 studies met 12, and 1 study met 10.

### Diagnostic Accuracy for the Prediction of Significant Fibrosis

**Figure 2:**
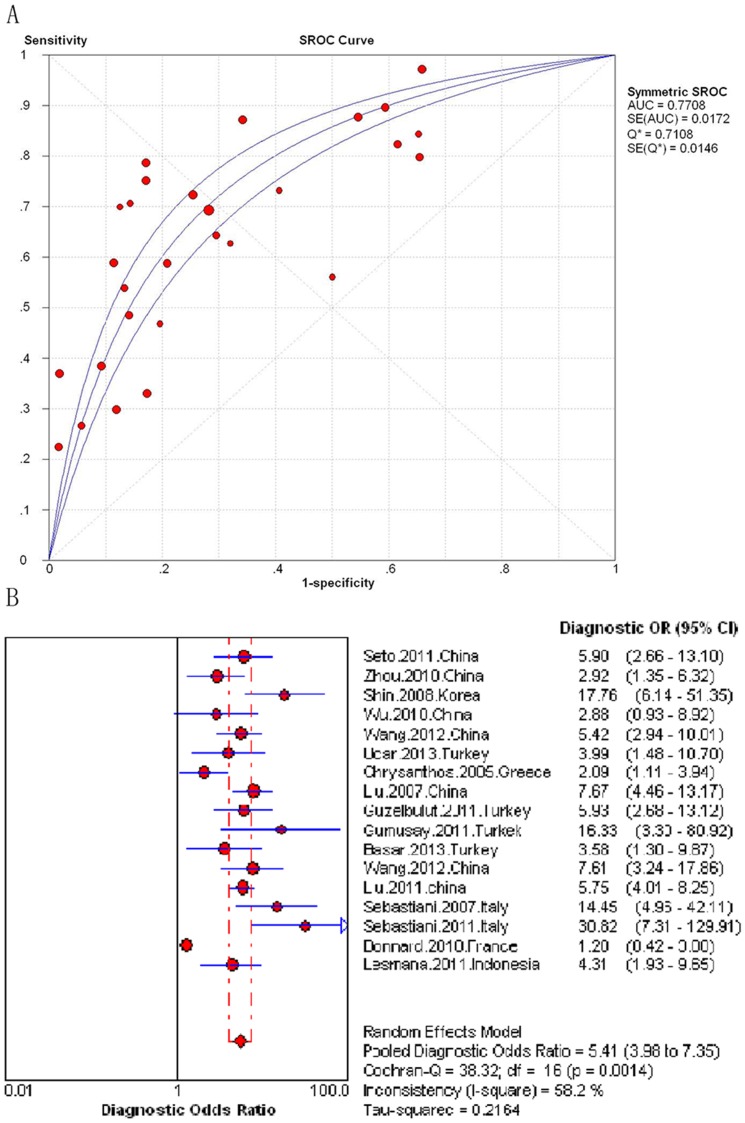
Meta-analysis of Hepatitis B-Related significant fibrosis. (A) SROC curve of the APRI; (B) Diagnostic odds ratio of the APRI.

In the seventeen studies assessing the APRI (N = 3,573), the AUROC curve ranged from 0.61 to 0.86. When combined, the area under the SROC curve was 0.77 08 (SE = 0.0172) (Figure 2A). The Pooled DOR was 5.41 (95% confidence interval [CI] 3.98–7.35) (Figure 2B). The Cochran-Q *and I^2^* value of all measures were 38.32 and 58.2%, indicating significant heterogeneity across the included studies (*P* = 0.001) (Figure 2B). The pooled sensitivities and specificities could not be assessed. Instead, the sensitivities and specificities of the APRI at various diagnostic thresholds in the seventeen studies are listed in Table 2. We used the meta-regression analysis to explore the heterogeneity of the APRI accuracy for detecting significant fibrosis, which was mainly affected by median age (*P* = 0.0211, see Text S4 for meta-regression). There was no significant correlation between other covariates and the DOR.

**Table 2:** Diagnostic Accuracy of APRI for the Prediction of Significant Fibrosis in Various Studies.

Author,Year	Cutoff	Sensitivity	Specificity	PPV	NPV	AUROC(95%CI)
Sebastiani, 2011	1.5	36%	98%	96%	53%	0.64(0.58–0.70)
Seto, 2011	0.5	89%	41%	42%	89%	0.71(0.63–0.80)
	1.5	29%	88%	55%	72%	
Bonnard, 2010	1	56%	50%	72%	33%	0.61(0.46–0.76)
Zhou, 2010	0.5	82%	38%	54%	71%	0.72
	1.5	48%	86%	75%	66%	
Shin, 2008	0.5	97%	34%	63%	91%	0.86(0.82–0.91)
	1	87%	66%	75%	82%	
	1.4	78%	83%	84%	77%	
	1.5	75%	83%	83%	74%	
	2	58%	89%	86%	65%	
Wu, 2010	0.5	84%	35%	47%	76%	0.71(0.59–0.83)
	1.5	46%	80%	63%	68%	
Wang, 2012	0.5	58%	79%	54%	82%	0.77(0.71–0.84)
Ucar, 2013	0.54	73%	59%	70%	63%	0.66
Chrysanthos, 2005	0.5	79%	35%	65%	53%	NA
	1.5	33%	83%	75%	45%	
Liu, 2007	0.4	72%	75%	54%	87%	0.77
Guzelbulut, 2011	0.5	87%	45%	36%	91%	0.78(0.72–0.84)
	1.5	38%	91%	60%	81%	
Lesmana, 2011	0.24	64%	70%	78%	54%	0.69(0.60–0.79)
Gumusay, 2011	0.7	70%	87%	54%	93%	0.82
Basar, 2013	0.43	62%	68%	80%	47%	0.67(0.55–0.79)
Wang, 2012	0.5	53%	86%	86%	56%	0.78
	1.5	22%	98%	95%	46%	
Liu, 2011	0.3	69%	71%	56%	82%	0.76(0.73–0.8)
Sebastiani, 2007	0.5	70%	85%	91%	58%	0.72(0.58–0.86)
	1.5	26%	94%	91%	38%	

APRI, aspartate aminotransferase-to-platelet ratio index; AUROC, area under the receiver operating characteristic; PPV, positive predictive value; NPV, negative predictive value; 95%CI, 95% confidence interval.

**Figure 3:**
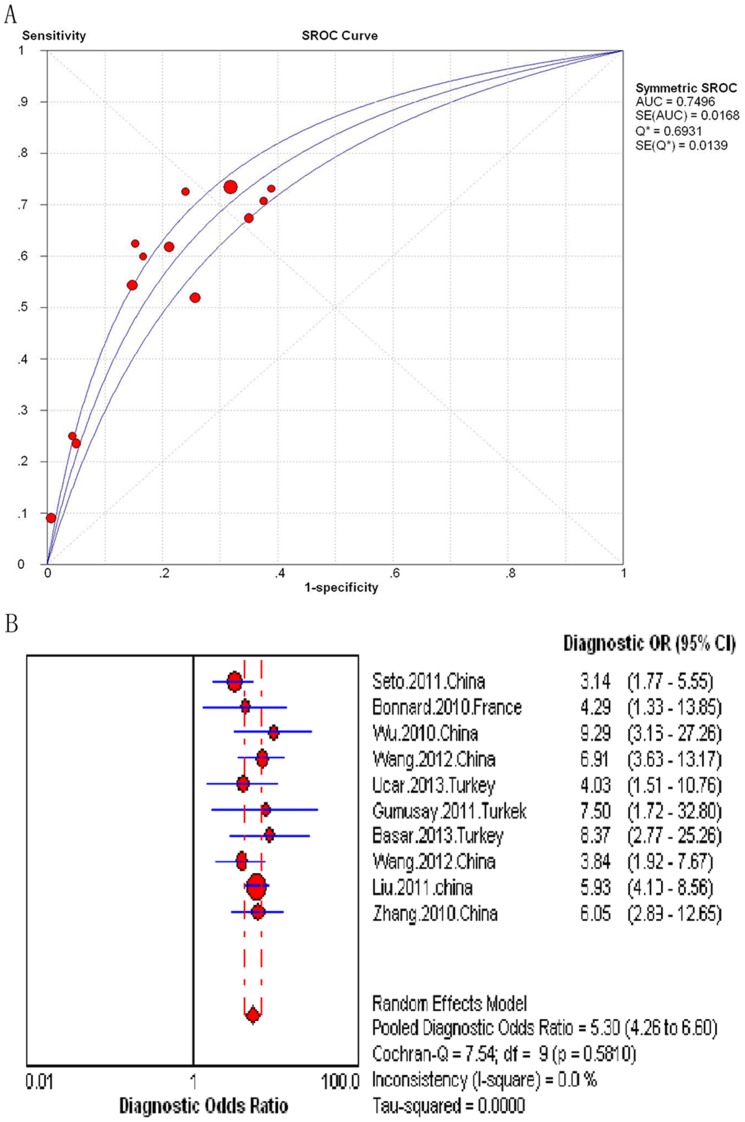
Meta-analysis of Hepatitis B-Related significant fibrosis. (A) SROC curve of the FIB-4; (B) Diagnostic odds ratio of the FIB-4.

In the ten studies assessing the FIB-4 for the prediction of significant Fibrosis (N = 1,996), the AUROC curve ranged from 0.69 to 0.77. When combined, the area under the SROC curve was 0.75 (SE = 0.0168) (Figure 3A). The summary DOR was 5.3 (95% CI 4.3–6.6), and the score of Cochran-Q is 7.54 (*P* = 0.581) (Figure 3B). The result from the analysis of the heterogeneity was statistically insignificant. The summary sensitivities and specificities of the FIB-4 were 65.8% (95% CI 62.4%–69.1%) and 73.6% (95% CI 70.8%–76.3%), respectively (Figure S1–S2).

**Figure 4:**
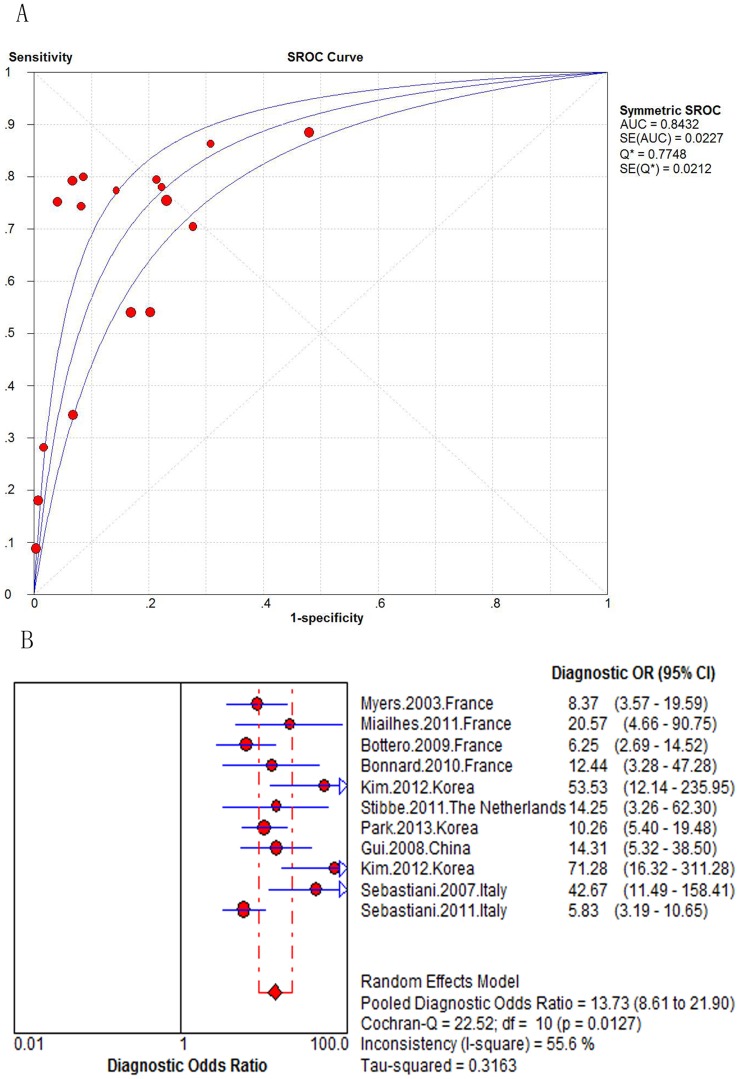
Meta-analysis of Hepatitis B-Related significant fibrosis. (A) SROC curve of the FibroTest; (B) Diagnostic odds ratio of the FibroTest.

The AUROC curve ranged from 0.69 to 0.90 in the 11 studies assessing the FibroTest (N = 1.640). When combined, the area under the SROC curve was 0.84 (SE = 0.0227) (Figure 4A). The summary DOR was 13.73 (95% CI 8.61–21.90), and the score of Cochran-Q is 22.52, indicating significant heterogeneity across the included studies (*P* = 0.0127) (Figure 4B). We didn't find the cause of the heterogeneity of FibroTest accuracy according to the predefined characteristics. But center description might affect heterogeneity beyond the predefined design (See Text S5).

### Diagnostic Accuracy for the Prediction of Cirrhosis

**Figure 5:**
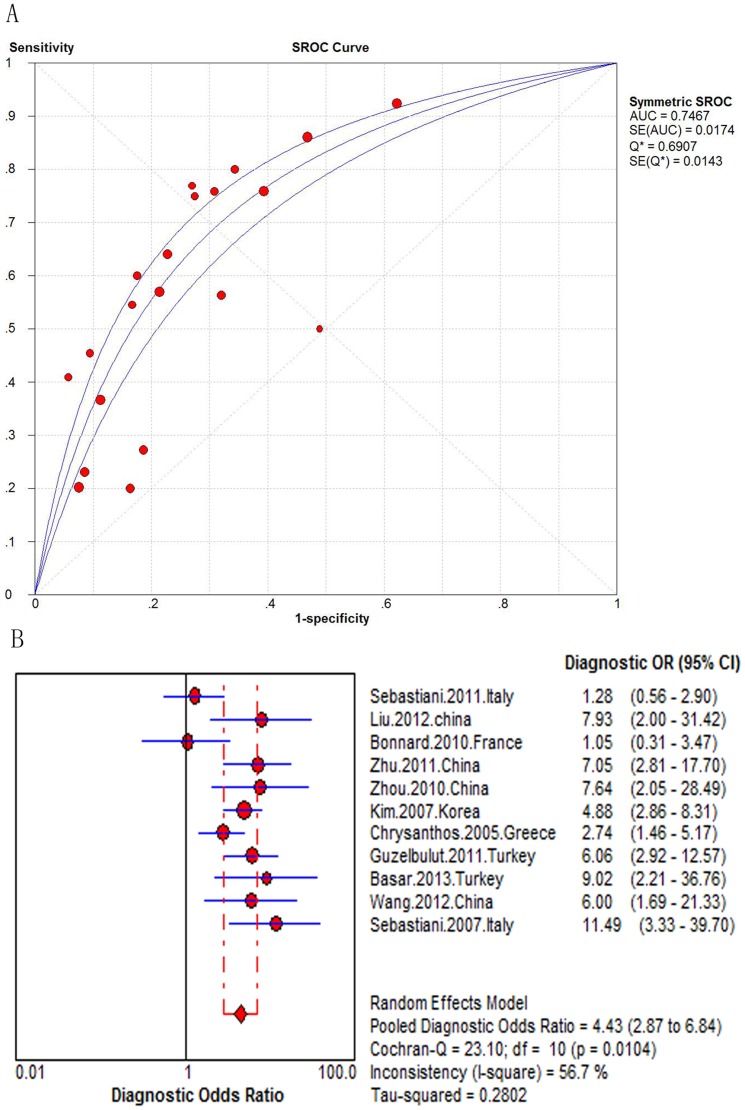
Meta-analysis of Hepatitis B-Related cirrhosis. (A) SROC curve of the APRI; (B) Diagnostic odds ratio of the APRI.

There were 11 studies on assessing the APRI for the predication of cirrhosis (N = 2,083). The AUROC curve of these studies ranged from 0.50 to 0.83. When combined, the area under the SROC curve was 0.75 (SE = 0.0174) (Figure 5A). The summary DOR was 4.4 (95% CI 2.9–6.8). The heterogeneity occurred in the meta-analysis for the twelve studies assessing the APRI for the predication of cirrhosis, which was statistically significant (Q = 23.10, *P* = 0.01; *I^2^* = 56.7%, Figure 5B). However, when we further conducted the meta-analysis at the different thresholds of <1.0, 1.0, and 2.0, we found that the heterogeneity wasn't statistically significant (Figure S3). The summary sensitivity and specificity of the APRI at different diagnostic thresholds are listed in Table 3.

**Table 3:** Summary Sensitivities and Specificities of the APRI at Different Diagnostic Thresholds for the Prediction of Cirrhosis.

Test Threshold	Number of Studies	SROC	Summary Sensitivity(95%CI)	Summary Specificity(95%CI)
<1.0	5(1,228)	0.76	84% (79%–88%)	54% (51%–58%)
1	6(1,471)	0.76	62% (55%–68%)	75% (72%–77%)
2	6(1,409)	0.79	29% (23%–35%)	89% (87%–91%)

APRI, aspartate aminotransferase-to-platelet ratio index; 95%CI, 95% confidence interval.

According to the meta-regression analysis, the heterogeneity of APRI accuracy for detecting cirrhosis was mainly affected by etiology (*P* = 0.0159) (See Text S6), whereas the other covariates were not significant. After excluding the only one study which included HBV/HDV-coinfected patients, the pooled DOR was 5.03 (95% CI 3.45–7.35) and heterogeneity was no longer significant (Q = 14.05, *P* = 0.1204; *I^2^* = 36.0%).(Figure S4) According to the meta-analysis, the pooled sensitivity and specificity were 60.9% (95% CI 55.0–66.6%) and 74.8% (72.4–77.1%), respectively (Figures S5–S6).

**Figure 6:**
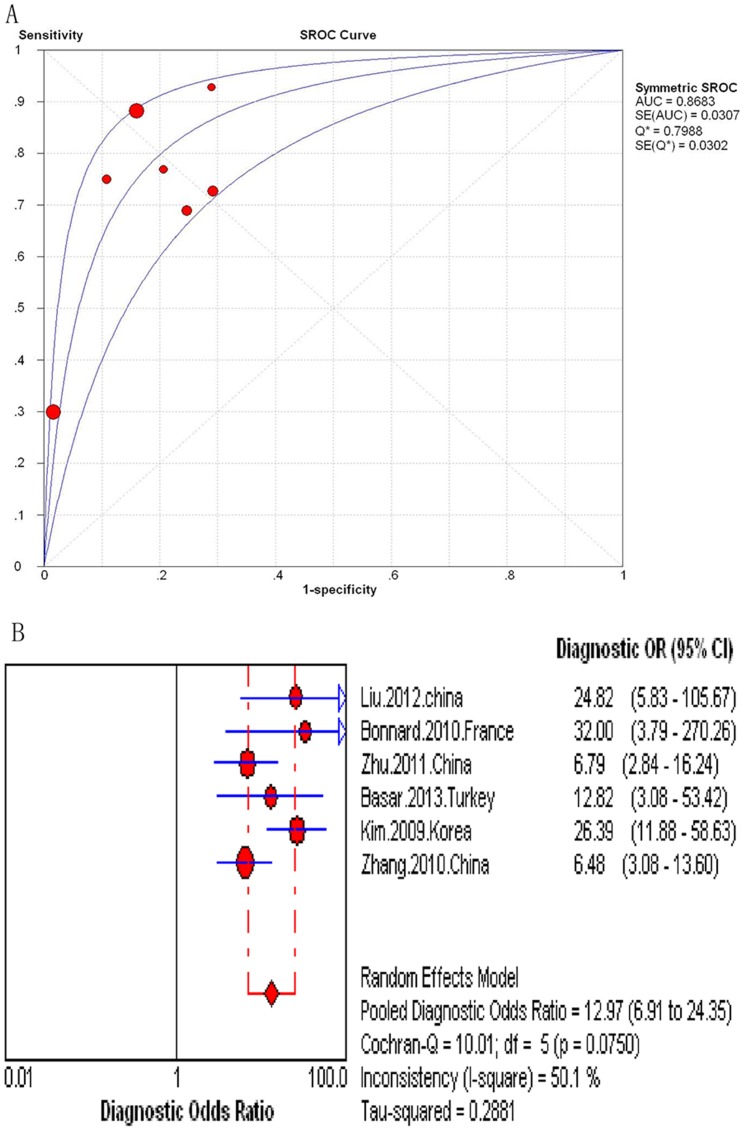
Meta-analysis of Hepatitis B-Related cirrhosis. (A) SROC curve of the FIB-4; (B) Diagnostic odds ratio of the FIB-4.

The AUROC curve in the six studies assessing the FIB-4 (N = 1,304) ranged from 0.74 to 0.93. When combined, the area under the SROC curve was 0.87 (SE = 0.0307) (Figure 6A). The summary DOR was 12.97 (95% CI 6.91–24.35) and the score of Cochran-Q is 10.01 (*P* = 0.07) (Figure 6B). The analysis showed that the heterogeneity was statistically insignificant. The summary sensitivities and specificities of the FIB-4 were 44.7% (95% CI 39.4%–50.2%) and 86.6% (95% CI 84.3%–88.7%), respectively (Figures S7–S8).

**Figure 7:**
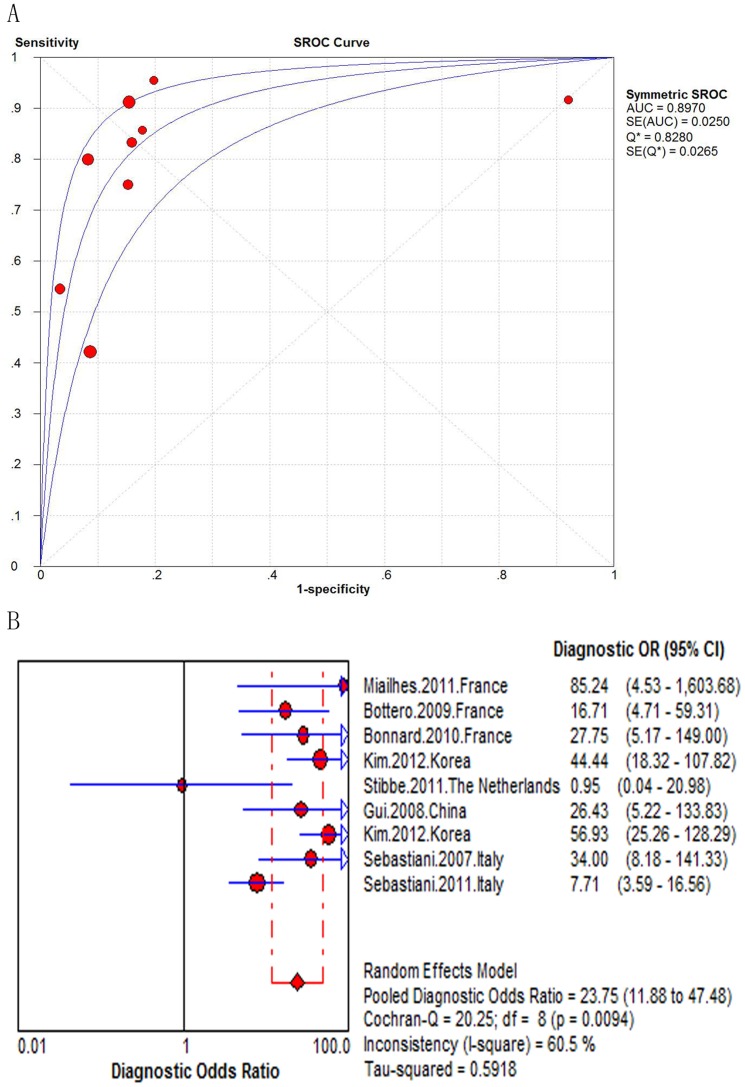
Meta-analysis of Hepatitis B-Related cirrhosis. (A) SROC curve of the FibroTest; (B) Diagnostic odds ratio of the FibroTest.

In the nine studies assessing the FibroTest (N = 1101), the AUROC curve ranged from 0.68 to 0.92. When combined, the area under the SROC curve was 0.90 (SE = 0.0250) (Figure 7A). The summary DOR was 23.75 (95% CI 11.88–47.48) and the score of Cochran-Q is 20.25 (*P* = 0.0094) (Figure 7B). The heterogeneity was statistically significant. The pooled sensitivities and specificities could not be assessed. Instead, the sensitivities and specificities of the FibroTest at various diagnostic thresholds in the nine studies are listed in Table 4.

**Table 4:** Diagnostic Accuracy of FibroTest for the Prediction of Significant Fibrosis and Cirrhosis in Various Studies.

Author,Year	Cutoff	Sensitivity	Specificity	PPV	NPV	AUROC(95%CI)
Significant Fibrosis						
Sebastiani, 2011	0.48	54%	83%	81%	57%	0.69(0.63–0.75)
Myers, 2003	0.2	89%	52%	43%	92%	0.78(0.74–0.82)
	0.4	54%	80%	52%	81%	
	0.6	34%	93%	68%	78%	
	0.8	18%	99%	92%	75%	
	1	8%	100%	100%	73%	
Miailhes, 2011	0.38	77%	85%	89%	72%	0.86(0.75–0.96)
Bottero, 2009	0.48	70%	72%	77%	65%	0.77(0.68–0.86)
Bonnard, 2010	0.37	78%	78%	89%	61%	0.79(0.66–0.91)
Kim, 2012	0.32	79%	93%	98%	45%	0.90(0.84–0.97)
Stibbe, 2011	0.31	86%	69%	70%	86%	NA
Park, 2013	0.32	75%	77%	93%	43%	NA
Gui, 2008	0.31	79%	79%	70%	86%	0.84(0.75–0.93)
	0.4	74%	92%	85%	85%	0.84(0.75–0.93)
	0.72	28%	98%	92%	68%	0.84(0.75–0.93)
Kim, 2012	0.31	75%	96%	98%	61%	0.9(0.85–0.94)
Sebastiani, 2007	F2	80%	91%	95%	68%	0.85(0.75–0.95)
Cirrhosis						
Sebastiani, 2011	0.75	42%	91%	51%	88%	0.68(0.63–0.73)
Miailhes, 2011	0.58	100%	81%	56%	100%	0.92(0.85–0.99)
Bottero, 2009	0.73	75%	85%	46%	95%	0.87(0.79–0.94)
Bonnard, 2010	0.5	86%	82%	60%	95%	0.85(0.74–0.96)
Kim, 2012	0.68	80%	92%	87%	87%	0.87(0.82–0.92)
Stibbe, 2011	0.75	100%	7%	11%	100%	NA
Gui, 2008	0.55	83%	84%	42%	97%	0.86(0.71–1.00)
Kim, 2012	0.67	91%	85%	85%	91%	0.88(0.83–0.94)
Sebastiani, 2007	F4	55%	97%	80%	89%	0.76(0.67–0.85)

According to the meta-regression analysis, the heterogeneity of FibroTest accuracy for detecting cirrhosis was mainly affected by sample size (*P* = 0.0385) and median age (*P* = 0.0436) (Text S7), whereas the other covariates were not significant.

### Publication Bias

**Figure 8:**
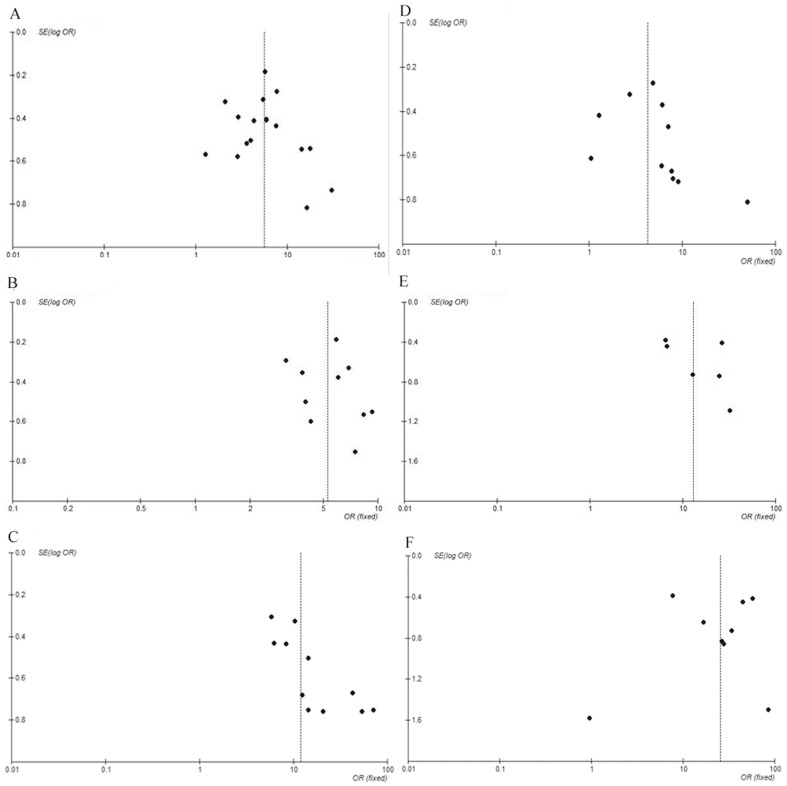
Funnel plot of publication bias. (A) APRI to predict significant fibrosis; (B) FIB-4 to predict significant fibrosis; (C) FibroTest to predict significant fibrosis; (D) APRI to predict cirrhosis; (E)FIB-4 to predict cirrhosis; (F) FibroTest to predict cirrhosis.

Funnel plots of these three markers for assessing possible publication bias are illustrated in Figure 8. Mild asymmetry was noted in the funnel plots of the FIB-4 and FibroTest.

## Discussion

Liver fibrosis progression is commonly found in HBV-infected patients. Cirrhosis develops in approximately one third of those cases, usually after an extensive period of time during which liver biochemical indices are found to be predominantly or even persistently abnormal [1]. Patients with significant fibrosis or cirrhosis should be considered for antiviral therapy, which can potentially reverse cirrhosis and reduce complications [60]. Considering the limitations and risks of biopsy, the researchers make persistent efforts in exploring some noninvasive markers in order to more accurately identify patients with significant fibrosis or cirrhosis. APRI, FIB-4 and FibroTest are such noninvasive markers gaining increasing acceptance in clinical practice. Those markers may reduce the need for liver biopsy and may help to monitor the efficacy of treatment [47].

In our systematic review, the diagnostic accuracy of the APRI, FIB-4 and FibroTest for HBV-related significant fibrosis and cirrhosis has been comprehensively evaluated and summarized on a large scale, and we confirmed the results of many individual studies. Our meta-analysis also included the description of multiple measures of test performance using confirmed meta-analytic techniques and formal assessment for publication bias and heterogeneity, as well as exploratory analysis. All results should be valid and reasonably reliable.

FibroTest had the best result in not only significant fibrosis but also cirrhosis. The area under the SROC curve of FibroTest is bigger and even reaches the standard of “better” on cirrhosis [15], and the summary sensitivity and specificity have reached 84% and 82%, respectively. A meta-analysis about HCV-infected patients showed that the area under the SROC curve of significant fibrosis and cirrhosis are 0.81 and 0.90 [12]. Evidently, the performance of FibroTest in evaluating HBV-related fibrosis is no worse than HCV-related. Therefore, FibroTest could be considered as a better marker in assessing fibrosis and cirrhosis of HBV-infected patients. The FibroTest, however, is calculated with alpha2 macroglobulin, alpha2 globulin (or haptoglobin), gamma globulin, apolipoprotein A1, GGT and total bilirubin [61]. Alpha2 macroglobulin and alpha2 globulin (or haptoglobin) are not routine clinical measurements, and those two indicators are not tested for patients in most hospitals. Furthermore they cost more than conventional indicators. Those factors may bring restrictions to the wider application of the FibroTest in clinical practice.

The calculation method of FIB-4 is simpler than that of FibroTest. The area under the SROC curve of FIB-4 predicting HBV-related significant fibrosis and cirrhosis are 0.75 and 0.87, respectively. FIB-4 also has a better performance of predicting fibrosis [7]. Its test items are easy to obtain in clinical practice, although its predictive results are not as good as FibroTest [11], [14]. APRI shows lower diagnostic accuracy than FibroTest and Fib-4 to identify HBV-related significant fibrosis and cirrhosis. It has been introduced to assess HBV-related fibrosis the earliest because of its simple and easy practice. Presently, APRI is widely utilized in identifying the degree of fibrosis and cirrhosis of patients with hepatitis C and hepatitis B, particularly in regions with limited healthcare resources. Some scholars argue that the calculation method of APRI did not consider the factor of spleen size [35]. If patients were grouped by spleen size, the performance of APRI in predicting HBV-related fibrosis would be improved. Our meta-analysis revealed that the area under the SROC curve of APRI was small and the accuracy of the evaluation of HBV-related fibrosis was poor. Our results showed similar performance of APRI for staging of significant fibrosis and cirrhosis [62].

Meta-regression method was convenient and reliable to screen the factors of heterogeneity. The strength of our study is that meta-regression analysis has been used to explore several factors that may be responsible for heterogeneity. Liver biopsy scoring systems and percentage of males emerged from many relevant factors to provide heterogeneity to summary test result on APRI to predict significant fibrosis [62]. On the other hand, etiology of cirrhosis was found to be significantly associated with the heterogeneity on APRI to predict cirrhosis. But the heterogeneity of the meta-analysis of the FIB-4 and FibroTest to predict significant fibrosis and cirrhosis was not statistically significant. FIB-4 and FibroTest to predict fibrosis had better consistency, and summary test results were reasonably reliable.

However, there are several limitations in our systematic review. Firstly, we only focused our analysis on those patients with HBV-related fibrosis, without distinguishing between HBeAg negative and positive cases, or considering the virus replication rate due to the limited number of publications. Secondly, we included studies published in English and Chinese languages only, so the language bias may influence the results to some extent. Lastly, Fibroscan, a widely noninvasive tool, was not considered in this meta-analysis, because our focus was to compare the serum markers calculated by biochemical examination.

In summary, the FibroTest has excellent diagnostic accuracy for the identification of HBV-related significant fibrosis and cirrhosis. But FibroTest is seldom applied in clinical practice as a result of expensive cost. FIB-4, a relatively moderate marker, has better summary diagnostic accuracy and could be measured and calculated relatively easily. Furthermore, APRI shows some limited value in identifying hepatitis B-related significant fibrosis and cirrhosis. All of them have their own advantages and disadvantages. Future studies of novel fibrosis markers are needed to demonstrate improved accuracy and cost-effectiveness compared with those simple, economical, and widely available indeces.

## Supporting Information

Figure S1 **Sensitivity of FIB-4 detecting significant fibrosis.**
(TIF)

Figure S2 **Specificity of FIB-4 detecting significant fibrosis.**
(TIF)

Figure S3 **DOR of APRI cirrhosis (subgroup).**
(TIF)

Figure S4 **DOR of APRI cirrhosis excluded patients with HBV and HDV coinfected.**
(TIF)

Figure S5 **Sensitivity of APRI cirrhosis excluded patients with HBV and HDV coinfected.**
(TIF)

Figure S6 **Specificity of APRI cirrhosis excluded patients with HBV and HDV coinfected.**
(TIF)

Figure S7 **Sensitivity of Fib-4 detecting cirrhosis.**
(TIF)

Figure S8 **Specificity of Fib-4 detecting cirrhosis.**
(TIF)

Checklist S1 **PRISMA checklist of items.**
(DOC)

Text S1 **Systematic review protocol.**
(DOC)

Text S2 **Search strategies.**
(DOC)

Text S3 **Quadas checklist.**
(DOC)

Text S4 **Meta-regression of APRI detecting significant fibrosis.**
(DOC)

Text S5 **Meta-regression of FibroTest detecting significant fibrosis.**
(RTF)

Text S6 **Meta-regression of APRI detecting cirrhosis.**
(RTF)

Text S7 **Meta-regression of FibroTest detecting cirrhosis.**
(RTF)

## References

[1] Custer B, Sullivan SD, Hazlet TK, Iloeje U, Veenstra DL, et al (2004) Global epidemiology of hepatitis B virus. J Clin Gastroenterol 38: S158–168. 15602165 10.1097/00004836-200411003-00008

[2] Yu MW, Hsu FC, Sheen IS, Chu CM, Lin DY, et al (1997) Prospective study of hepatocellular carcinoma and liver cirrhosis in asymptomatic chronic hepatitis B virus carriers. Am J Epidemiol 145: 1039–1047. 9169913 10.1093/oxfordjournals.aje.a009060

[3] Gumusay O, Ozenirler S, Atak A, Sonmez C, Ozkan S, et al (2013) Diagnostic potential of serum direct markers and non-invasive fibrosis models in patients with chronic hepatitis B. Hepatol Res. 43: 228–237. 10.1111/j.1872-034X.2012.01057.x 22734888

[4] Liaw YF, Sung JJ, Chow WC, Farrell G, Lee CZ, et al (2004) Lamivudine for patients with chronic hepatitis B and advanced liver disease. N Engl J Med 351: 1521–1531. 15470215 10.1056/NEJMoa033364

[5] Bravo AA, Sheth SG, Chopra S (2001) Liver biopsy. N Engl J Med 344: 495–500. 11172192 10.1056/NEJM200102153440706

[6] Colloredo G, Guido M, Sonzogni A, Leandro G (2003) Impact of liver biopsy size on histological evaluation of chronic viral hepatitis: the smaller the sample, the milder the disease. J Hepatol 39: 239–244. 12873821 10.1016/s0168-8278(03)00191-0

[7] Kim BK, Kim do Y, Park JY, Ahn SH, Chon CY, et al (2010) Validation of FIB-4 and comparison with other simple noninvasive indices for predicting liver fibrosis and cirrhosis in hepatitis B virus-infected patients. Liver Int 30: 546–553. 20074094 10.1111/j.1478-3231.2009.02192.x

[8] Sterling RK, Wegelin JA, Smith PG, Stravitz RT, Luketic VA, et al (2010) Similar progression of fibrosis between HIV/HCV-infected and HCV-infected patients: Analysis of paired liver biopsy samples. Clin Gastroenterol Hepatol 8: 1070–1076. 20728569 10.1016/j.cgh.2010.08.004 PMC2997143

[9] Poynard T, Morra R, Halfon P, Castera L, Ratziu V, et al (2007) Meta-analyses of FibroTest diagnostic value in chronic liver disease. BMC Gastroenterol 7: 40. 17937811 10.1186/1471-230X-7-40 PMC2175505

[10] Corradi F, Piscaglia F, Flori S, D'Errico-Grigioni A, Vasuri F, et al (2009) Assessment of liver fibrosis in transplant recipients with recurrent HCV infection: usefulness of transient elastography. Dig Liver Dis 41: 217–225. 18672413 10.1016/j.dld.2008.06.009

[11] Amorim TG, Staub GJ, Lazzarotto C, Silva AP, Manes J, et al (2012) Validation and comparison of simple noninvasive models for the prediction of liver fibrosis in chronic hepatitis C. Ann Hepatol. 11: 855–861. 23109448

[12] Shaheen AA, Wan AF, Myers RP (2007) FibroTest and FibroScan for the prediction of hepatitis C-related fibrosis: a systematic review of diagnostic test accuracy. Am J Gastroenterol 102: 2589–2600. 17850410 10.1111/j.1572-0241.2007.01466.x

[13] Lin ZH, Xin YN, Dong QJ, Wang Q, Jiang XJ, et al (2011) Performance of the aspartate aminotransferase-to-platelet ratio index for the staging of hepatitis C-related fibrosis: an updated meta-analysis. Hepatology 53: 726–736. 21319189 10.1002/hep.24105

[14] Martinez SM, Fernandez-Varo G, Gonzalez P, Sampson E, Bruguera M, et al (2011) Assessment of liver fibrosis before and after antiviral therapy by different serum marker panels in patients with chronic hepatitis C. Aliment Pharmacol Ther. 33: 138–148. 10.1111/j.1365-2036.2010.04500.x 21083589

[15] Deeks JJ (2001) Systematic reviews in health care: Systematic reviews of evaluations of diagnostic and screening tests. BMJ 323: 157–162. 11463691 10.1136/bmj.323.7305.157 PMC1120791

[16] Chrysanthos NV, Papatheodoridis GV, Savvas S, Kafiri G, Petraki K, et al (2006) Aspartate aminotransferase to platelet ratio index for fibrosis evaluation in chronic viral hepatitis. Eur J Gastroenterol Hepatol 18: 389–396. 16538110 10.1097/00042737-200604000-00012

[17] Wang H, Xue L, Yan R, Zhou Y, Wang MS, et al (2013) Comparison of FIB-4 and APRI in Chinese HBV-infected patients with persistently normal ALT and mildly elevated ALT. J Viral Hepat 20: e3–10. 23490387 10.1111/jvh.12010

[18] Moher D, Liberati A, Tetzlaff J, Altman DG (2009) Preferred reporting items for systematic reviews and meta-analyses: the PRISMA statement. PLoS Med 6: e1000097. 19621072 10.1371/journal.pmed.1000097 PMC2707599

[19] Whiting P, Rutjes AW, Reitsma JB, Bossuyt PM, Kleijnen J (2003) The development of QUADAS: a tool for the quality assessment of studies of diagnostic accuracy included in systematic reviews. BMC Med Res Methodol 3: 25. 14606960 10.1186/1471-2288-3-25 PMC305345

[20] Group TFMCS (1994) Intraobserver and interobserver variations in liver biopsy interpretation in patients with chronic hepatitis C. The French METAVIR Cooperative Study Group. Hepatology 20: 15–20. 8020885

[21] Batts KP, Ludwig J (1995) Chronic hepatitis. An update on terminology and reporting. Am J Surg Pathol 19: 1409–1417. 7503362 10.1097/00000478-199512000-00007

[22] Scheuer PJ (1991) Classification of chronic viral hepatitis: a need for reassessment. J Hepatol 13: 372–374. 1808228 10.1016/0168-8278(91)90084-o

[23] Ishak K, Baptista A, Bianchi L, Callea F, De Groote J, et al (1995) Histological grading and staging of chronic hepatitis. J Hepatol 22: 696–699. 7560864 10.1016/0168-8278(95)80226-6

[24] Strader DB, Wright T, Thomas DL, Seeff LB (2004) Diagnosis, management, and treatment of hepatitis C. Hepatology. 39: 1147–1171. 10.1002/hep.20119 15057920

[25] Walter SD (2002) Properties of the summary receiver operating characteristic (SROC) curve for diagnostic test data. Stat Med 21: 1237–1256. 12111876 10.1002/sim.1099

[26] Moses LE, Shapiro D, Littenberg B (1993) Combining independent studies of a diagnostic test into a summary ROC curve: data-analytic approaches and some additional considerations. Stat Med 12: 1293–1316. 8210827 10.1002/sim.4780121403

[27] Dukic V, Gatsonis C (2003) Meta-analysis of diagnostic test accuracy assessment studies with varying number of thresholds. Biometrics 59: 936–946. 14969472 10.1111/j.0006-341x.2003.00108.x PMC10425262

[28] DerSimonian R, Laird N (1986) Meta-analysis in clinical trials. Control Clin Trials 7: 177–188. 3802833 10.1016/0197-2456(86)90046-2

[29] Doust JA, Glasziou PP, Pietrzak E, Dobson AJ (2004) A systematic review of the diagnostic accuracy of natriuretic peptides for heart failure. Arch Intern Med 164: 1978–1984. 15477431 10.1001/archinte.164.18.1978

[30] Reitsma JB, Glas AS, Rutjes AW, Scholten RJ, Bossuyt PM, et al (2005) Bivariate analysis of sensitivity and specificity produces informative summary measures in diagnostic reviews. J Clin Epidemiol 58: 982–990. 16168343 10.1016/j.jclinepi.2005.02.022

[31] Higgins JP, Thompson SG, Deeks JJ, Altman DG (2003) Measuring inconsistency in meta-analyses. BMJ 327: 557–560. 12958120 10.1136/bmj.327.7414.557 PMC192859

[32] Deeks JJ, Macaskill P, Irwig L (2005) The performance of tests of publication bias and other sample size effects in systematic reviews of diagnostic test accuracy was assessed. J Clin Epidemiol 58: 882–893. 16085191 10.1016/j.jclinepi.2005.01.016

[33] Zhou K, Gao CF, Zhao YP, Liu HL, Zheng RD, et al (2010) Simpler score of routine laboratory tests predicts liver fibrosis in patients with chronic hepatitis B. J Gastroenterol Hepatol. 25: 1569–1577. 10.1111/j.1440-1746.2010.06383.x 20796157

[34] Shin WG, Park SH, Jang MK, Hahn TH, Kim JB, et al (2008) Aspartate aminotransferase to platelet ratio index (APRI) can predict liver fibrosis in chronic hepatitis B. Dig Liver Dis. 40: 267–274. 10.1016/j.dld.2007.10.011 18055281

[35] Kim BK, Kim SA, Park YN, Cheong JY, Kim HS, et al (2007) Noninvasive models to predict liver cirrhosis in patients with chronic hepatitis B. Liver Int. 27: 969–976. 10.1111/j.1478-3231.2007.01519.x 17696936

[36] Hongbo L, Xiaohui L, Hong K, Wei W, Yong Z (2007) Assessing routine and serum markers of liver fibrosis in CHB patients using parallel and serial interpretation. Clin Biochem 40: 562–566. 17362902 10.1016/j.clinbiochem.2007.01.022

[37] Guzelbulut F, Sezikli M, Akkan-Cetinkaya Z, Yasar B, Ozkara S, et al (2012) AST-platelet ratio index in the prediction of significant fibrosis and cirrhosis in patients with chronic hepatitis B. Turk J Gastroenterol. 23: 353–358. 10.4318/tjg.2012.0348 22965506

[38] Lesmana CR, Salim S, Hasan I, Sulaiman AS, Gani RA, et al (2011) Diagnostic accuracy of transient elastography (FibroScan) versus the aspartate transaminase to platelet ratio index in assessing liver fibrosis in chronic hepatitis B: the role in primary care setting. J Clin Pathol 64: 916–920. 21670074 10.1136/jclinpath-2011-200044

[39] Seto WK, Lee CF, Lai CL, Ip PP, Fong DY, et al (2011) A new model using routinely available clinical parameters to predict significant liver fibrosis in chronic hepatitis B. PLoS One. 6: e23077. 10.1371/journal.pone.0023077 PMC3154931 21853071

[40] Liu XD, Wu JL, Liang J, Zhang T, Sheng QS (2012) Globulin-platelet model predicts minimal fibrosis and cirrhosis in chronic hepatitis B virus infected patients. World J Gastroenterol 18: 2784–2792. 22719186 10.3748/wjg.v18.i22.2784 PMC3374981

[41] Zhu X, Wang LC, Chen EQ, Chen XB, Chen LY, et al (2011) Prospective evaluation of FibroScan for the diagnosis of hepatic fibrosis compared with liver biopsy/AST platelet ratio index and FIB-4 in patients with chronic HBV infection. Dig Dis Sci 56: 2742–2749. 21399926 10.1007/s10620-011-1659-1

[42] Wu SD, Wang JY, Li L (2010) Staging of liver fibrosis in chronic hepatitis B patients with a composite predictive model: a comparative study. World J Gastroenterol 16: 501–507. 20101779 10.3748/wjg.v16.i4.501 PMC2811806

[43] Wang Y, Xu MY, Zheng RD, Xian JC, Xu HT, et al (2013) Prediction of significant fibrosis and cirrhosis in hepatitis B e-antigen negative patients with chronic hepatitis B using routine parameters. Hepatol Res 43: 441–451. 23006433 10.1111/j.1872-034X.2012.01094.x

[44] Ucar F, Sezer S, Ginis Z, Ozturk G, Albayrak A, et al.. (2013) APRI, the FIB-4 score, and Forn's index have noninvasive diagnostic value for liver fibrosis in patients with chronic hepatitis B. Eur J Gastroenterol Hepatol. 10.1097/MEG.0b013e32835fd699 23510962

[45] Basar O, Yimaz B, Ekiz F, Ginis Z, Altinbas A, et al (2013) Non-invasive tests in prediction of liver fibrosis in chronic hepatitis B and comparison with post-antiviral treatment results. Clin Res Hepatol Gastroenterol 37: 152–158. 23391746 10.1016/j.clinre.2012.07.003

[46] Liu HB, Zhou JP, Zhang Y, Lv XH, Wang W (2011) Prediction on liver fibrosis using different APRI thresholds when patient age is a categorical marker in patients with chronic hepatitis B. Clin Chim Acta. 412: 33–37. 10.1016/j.cca.2010.08.032 20828546

[47] Sebastiani G, Vario A, Guido M, Alberti A (2007) Sequential algorithms combining non-invasive markers and biopsy for the assessment of liver fibrosis in chronic hepatitis B. World J Gastroenterol. 13: 525–531. 10.3748/wjg.v13.i4.525 PMC4065973 17278217

[48] Bonnard P, Sombie R, Lescure FX, Bougouma A, Guiard-Schmid JB, et al (2010) Comparison of elastography, serum marker scores, and histology for the assessment of liver fibrosis in hepatitis B virus (HBV)-infected patients in Burkina Faso. Am J Trop Med Hyg 82: 454–458. 20207872 10.4269/ajtmh.2010.09-0088 PMC2829908

[49] Sebastiani G, Castera L, Halfon P, Pol S, Mangia A, et al (2011) The impact of liver disease aetiology and the stages of hepatic fibrosis on the performance of non-invasive fibrosis biomarkers: an international study of 2411 cases. Alimentary Pharmacology & Therapeutics 34: 1202–1216. 21981787 10.1111/j.1365-2036.2011.04861.x

[50] Zhang YF, Shi H, Chen LB, Xu QH (2010) [Value of FIB-4 for the diagnosis of liver fibrosis in chronic hepatitis B]. Zhonghua Shi Yan He Lin Chuang Bing Du Xue Za Zhi 24: 215–217. 21186531

[51] Myers RP, Tainturier MH, Ratziu V, Piton A, Thibault V, et al (2003) Prediction of liver histological lesions with biochemical markers in patients with chronic hepatitis B. J Hepatol. 39: 222–230. 10.1016/s0168-8278(03)00171-5 12873819

[52] Miailhes P, Pradat P, Chevallier M, Lacombe K, Bailly F, et al (2011) Proficiency of transient elastography compared to liver biopsy for the assessment of fibrosis in HIV/HBV-coinfected patients. J Viral Hepat 18: 61–69. 20196798 10.1111/j.1365-2893.2010.01275.x

[53] Bottero J, Lacombe K, Guechot J, Serfaty L, Miailhes P, et al (2009) Performance of 11 biomarkers for liver fibrosis assessment in HIV/HBV co-infected patients. J Hepatol 50: 1074–1083. 19398234 10.1016/j.jhep.2009.01.022

[54] Kim BK, Kim SU, Kim HS, Park JY, Ahn SH, et al (2012) Prospective validation of FibroTest in comparison with liver stiffness for predicting liver fibrosis in Asian subjects with chronic hepatitis B. PLoS One. 7: e35825. 10.1371/journal.pone.0035825 PMC3335013 22536445

[55] Stibbe KJ, Verveer C, Francke J, Hansen BE, Zondervan PE, et al (2011) Comparison of non-invasive assessment to diagnose liver fibrosis in chronic hepatitis B and C patients. Scand J Gastroenterol 46: 962–972. 21623677 10.3109/00365521.2011.574725

[56] Park MS, Kim BK, Cheong JY, Kim DJ, Park JY, et al (2013) Discordance between liver biopsy and FibroTest in assessing liver fibrosis in chronic hepatitis B. PLoS One. 8: e55759. 10.1371/journal.pone.0055759 PMC3566034 23405210

[57] Gui HL, Xie Q, Wang H (2008) [FibroTest-ActiTest for predicting liver fibrosis and inflammatory activity in Chinese patients with chronic hepatitis B]. Zhonghua Gan Zang Bing Za Zhi 16: 897–901. 19105932

[58] Kim BK, Kim HS, Park JY, Kim do Y, Ahn SH, et al (2012) Prospective validation of ELF test in comparison with Fibroscan and FibroTest to predict liver fibrosis in Asian subjects with chronic hepatitis B. PLoS One. 7: e41964. 10.1371/journal.pone.0041964 PMC3407050 22848675

[59] Sebastiani G, Castera L, Halfon P, Pol S, Mangia A, et al (2011) The impact of liver disease aetiology and the stages of hepatic fibrosis on the performance of non-invasive fibrosis biomarkers: an international study of 2411 cases. Aliment Pharmacol Ther 34: 1202–1216. 21981787 10.1111/j.1365-2036.2011.04861.x

[60] Lavanchy D (2004) Hepatitis B virus epidemiology, disease burden, treatment, and current and emerging prevention and control measures. J Viral Hepat 11: 97–107. 14996343 10.1046/j.1365-2893.2003.00487.x

[61] Imbert-Bismut F, Ratziu V, Pieroni L, Charlotte F, Benhamou Y, et al (2001) Biochemical markers of liver fibrosis in patients with hepatitis C virus infection: a prospective study. Lancet 357: 1069–1075. 11297957 10.1016/S0140-6736(00)04258-6

[62] Jin W, Lin Z, Xin Y, Jiang X, Dong Q, et al (2012) Diagnostic accuracy of the aspartate aminotransferase-to-platelet ratio index for the prediction of hepatitis B-related fibrosis: a leading meta-analysis. BMC Gastroenterol 12: 14. 22333407 10.1186/1471-230X-12-14 PMC3306191

