# Supplementary figures and images for: The Effectiveness of Noninvasive Biomarkers to Predict Hepatitis B-Related Significant Fibrosis and Cirrhosis: A Systematic Review and Meta-Analysis of Diagnostic Test Accuracy
Source: PLoS One. 2014 Jun 25;9(6):e100182. doi: 10.1371/journal.pone.0100182 (PMC4070977; doi:10.1371/journal.pone.0100182)

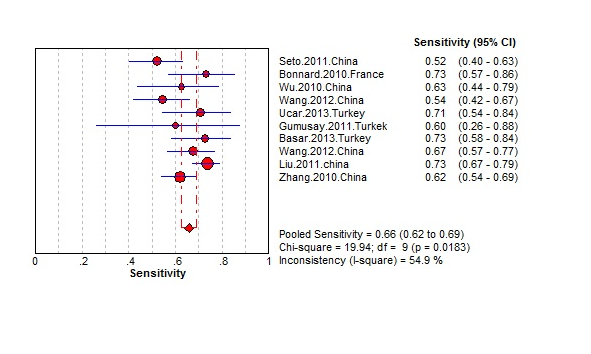

Supplement: Figure S1 — Sensitivity of FIB-4 detecting significant fibrosis. (TIF) [file pone.0100182.s001.tif]

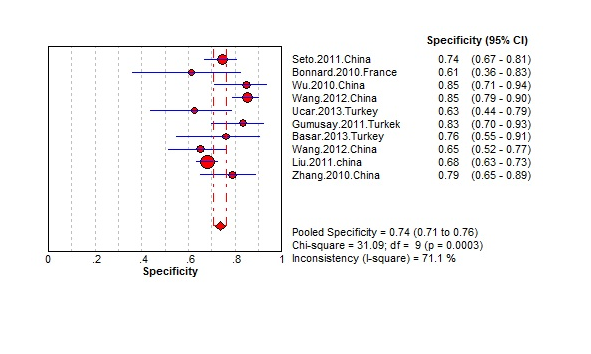

Supplement: Figure S2 — Specificity of FIB-4 detecting significant fibrosis. (TIF) [file pone.0100182.s002.tif]

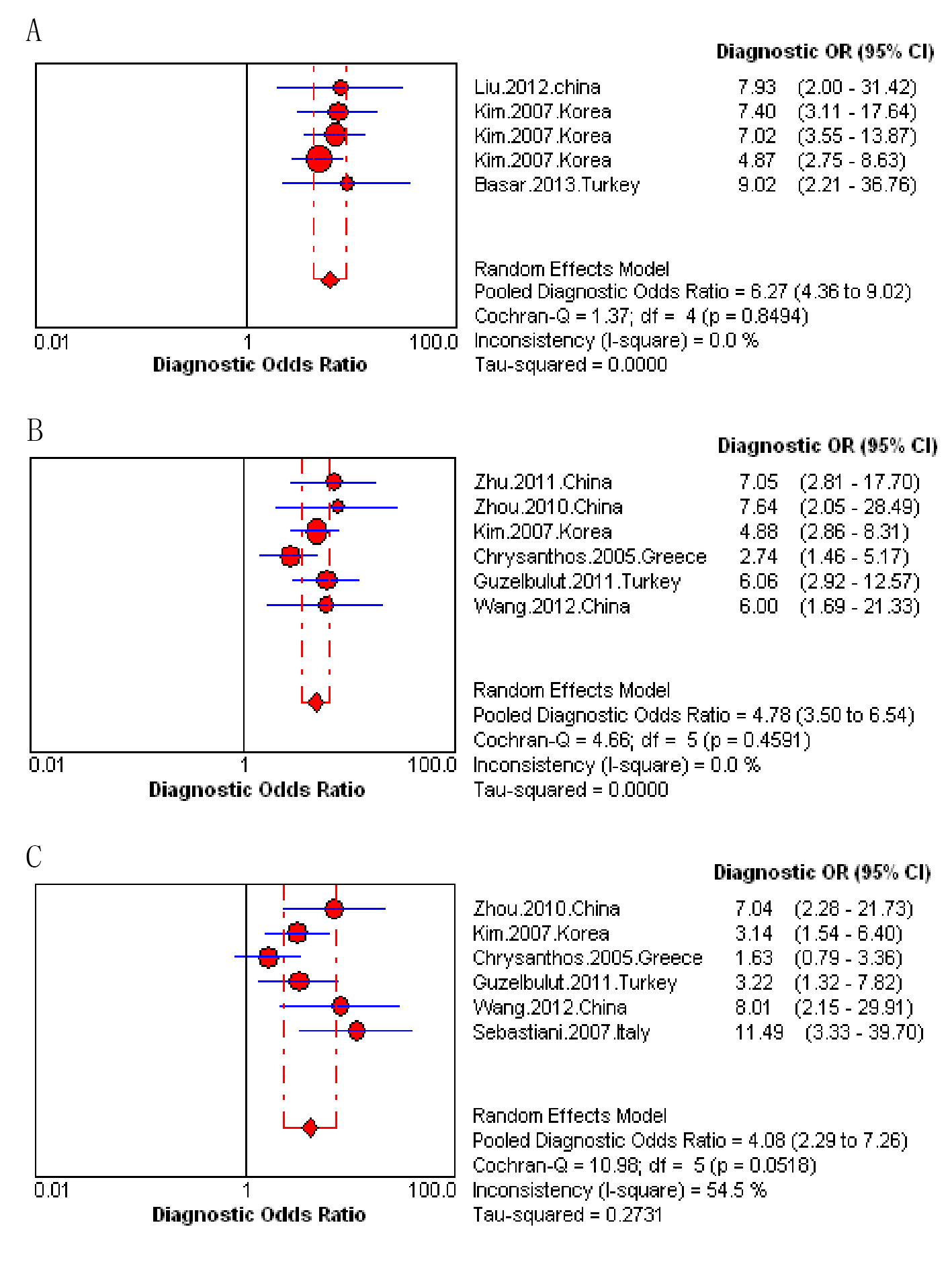

Supplement: Figure S3 — DOR of APRI cirrhosis (subgroup). (TIF) [file pone.0100182.s003.tif]

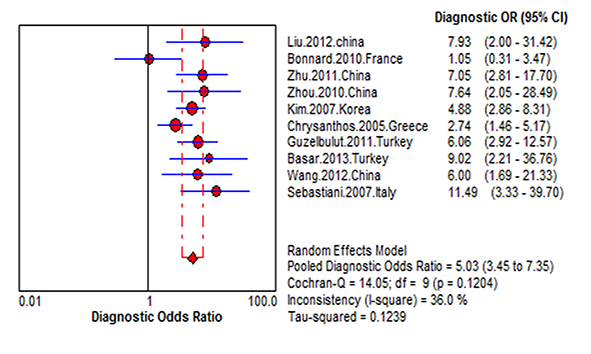

Supplement: Figure S4 — DOR of APRI cirrhosis excluded patients with HBV and HDV coinfected. (TIF) [file pone.0100182.s004.tif]

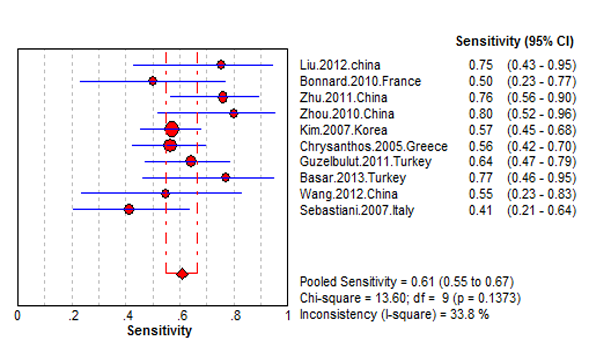

Supplement: Figure S5 — Sensitivity of APRI cirrhosis excluded patients with HBV and HDV coinfected. (TIF) [file pone.0100182.s005.tif]

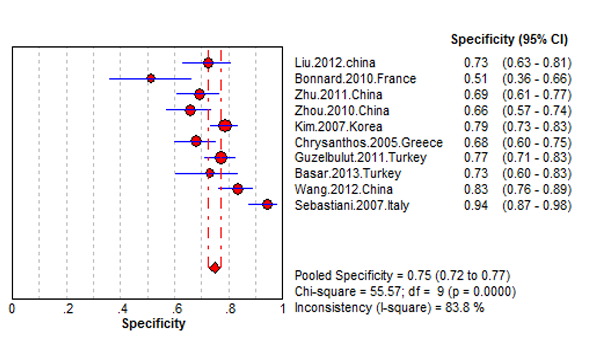

Supplement: Figure S6 — Specificity of APRI cirrhosis excluded patients with HBV and HDV coinfected. (TIF) [file pone.0100182.s006.tif]

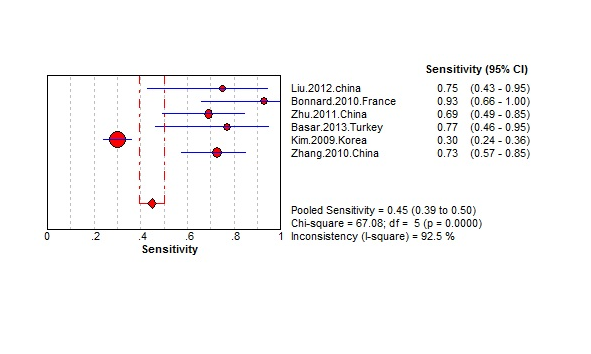

Supplement: Figure S7 — Sensitivity of Fib-4 detecting cirrhosis. (TIF) [file pone.0100182.s007.tif]

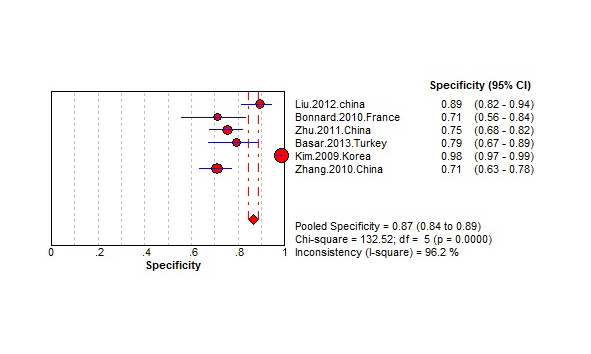

Supplement: Figure S8 — Specificity of Fib-4 detecting cirrhosis. (TIF) [file pone.0100182.s008.tif]
