# Supplementary material for: The Effectiveness of Noninvasive Biomarkers to Predict Hepatitis B-Related Significant Fibrosis and Cirrhosis: A Systematic Review and Meta-Analysis of Diagnostic Test Accuracy
Source: PLoS One. 2014 Jun 25;9(6):e100182. doi: 10.1371/journal.pone.0100182 (PMC4070977; doi:10.1371/journal.pone.0100182)
Supplement: Text S1 — Systematic review protocol. (DOC) [file pone.0100182.s010.doc]

**Protocol**

# The Effectiveness of Noninvasive Biomarkers to Predict Hepatitis B-Related Significant Fibrosis and Cirrhosis: A Systematic Review and Meta-Analysis of Diagnostic Test Accuracy

Hong-Bo Liu^1^ (hbliu@mail.cmu.edu.cn)

^1^Public Health of School, China Medical University, Shenyang, PR China;

**Summary**

***Background***

Noninvasive biomarkers have been developed to predict hepatitis B virus (HBV)-related fibrosis owing to the limitations of liver biopsy. But these biomarkers were initially derived from evaluation of hepatitis C virus (HCV)-related fibrosis, their accuracy among HBV-related patients was always disputed. The aim of this study was to systematically assess the effectiveness of these biomarkers for predicting HBV-related fibrosis.

***Review objectives***

To summarise the existing literature and assess the effectiveness of noninvasive biomarkers including aspartate aminotransferase-to-platelet ratio index (APRI), fibrosis index based on the 4 factors (FIB-4) and FibroTest for predicting HBV-related fibrosis.

***Population***

Adults who have a diagnosis of HBV-related disease (ICD10 B16 and B18), participating in studies published in a peer-reviewed journal up to 2013, and where Liver biopsy, the gold standard, is used to assess the level of fibrosis. All patients in these literature were predicted HBV-related fibrosis according to APRI and/or FIB-4 and/or FibroTest.

***Outcomes***

ⅰ)overall prevalence of significant fibrosis and cirrhosis; or ii) Area under the SROC curve，Diagnostic Odds Ratio, Summary Sensitivities and Specificities, and meta-regression technique

***Study design***

Observational studies conducting appropriate univariable or multivariable analyses

***Background***

Chronic infection with the hepatitis B virus (HBV) is an important global health problem. Approximately 350 million people are chronically infected with the hepatitis B virus worldwide, especially in developing countries, 25% of whom will die from long term sequelae, such as cirrhosis, liver failure and hepatocellular carcinoma, resulting in 600,000 to one million deaths annually. Assessment of liver significant ﬁbrosis is critical to constitute logical clinical practice. It could be great help for a doctor to determine patients’ suitability and the optimal time for antiviral therapy prevent from abusing drug . In addition , early prediction of cirrhosis is beneficial to reduce complications in patients with chronic viral hepatitis.

Liver biopsy, an invasive technique, is the gold standard for the assessment of fibrosis. It has several disadvantages, such as patients’ reluctance, pain, hemoperitoneum, and pneumothorax, etc. In addition, its accuracy in assessing ﬁbrsis is questionable because of sampling errors and intra- and interobserver variations. Therefore, many people are beginning to realize the importance of prediction of liver fibrosis by some noninvasive methods.

Aspartate aminotransferase-to-platelet ratio index (APRI), fibrosis index based on the 4 factors (FIB-4) and FibroTest are noninvise biomarkers predicting liver fibrosis based on routinely available clinical parameters. They were initially used in Western populations with hepatitis C virus (HCV) or HCV/ human immunodeficiency virus (HIV) co-infection and had good performance. Therefore, the researchers were conducting these markers to predict significant fibrosis and cirrhosis among HBV-related patients. APRI was firstly used to predict significant fibrosis or cirrhosis in patients with HBeAg-negative chronic hepatitis B. [8,9] They found APRI was significantly associated with the fibrosis. FIB-4 and FibroTest were successively used to predict HBV-related fibrosis.

However, due to these markers initially derived from evaluation of HCV-related fibrosis, their accuracy among HBV-related patients was always disputed by the researchers. Some scholars indicate that all of them are noninvasive markers to predict significant fibrosis or cirrhosis among HBV-related patients and potentially could be used to decrease the number of liver biopsies. [6, 9] But others maintain that these markers were not applied to evaluation of HBV-related fibrosis because of the small AUROC curve. [6, 10] Therefore, we conducted this meta-analysis to assess the performance of these combined biomarkers for prediction of significant fibrosis and cirrhosis among hepatitis B-related patients. It could provide the basis for future research and clinical application.

***Methods***

***Search strategy***

Systematic methods will be used to identify relevant studies, assess study eligibility for inclusion and evaluate study quality. A search will be undertaken to locate all studies published up to June 2013 predicting significant fibrosis or cirrhosis by APRI, FIB-4, and FibroTest in HBV infected patients. One researcher (Xu) will develop the search strategy on the online databases of MEDLINE, EMBASE and the Cochrane Library (01/2003-04/2013). Additional studies will be identified by reviewing the reference lists of relevant studies identified from the search and by a manual search to identify more recent studies that have cited an older, relevant study. EndNote software will be used to manage the references.

***Study Eligibility***

Adults who have a diagnosis of HBV-related disease (ICD10 B16 and B18), participating in studies published in a peer-reviewed journal up to 2013, and where Liver biopsy, the gold standard, is used to assess the level of fibrosis. Preliminary independent screening of the titles and abstracts obtained from the database searches will be carried out by two researchers (XYX and RXS). Initial screening of titles will be carried out to remove obviously irrelevant papers. However, from a preliminary scoping review by XYX, the early pilot searches recovered studies that, although they conducted analyses by HK, did not always mention this in the abstract or title. Selected abstracts will then be screened and a subset of studies will be selected for further review and the full article obtained.

Two researchers (WSA and YHZ) will then independently assess the selected full papers for eligibility according to the study-eligibility criteria detailed above. Any disagreements at any of the screening stages will be resolved by discussion between the two reviewers in the first instance. If agreement cannot be reached, then a third reviewer (HBL) will independently review the title, abstract or full paper, as appropriate, and a majority decision will be taken on inclusion/exclusion.

***Data Extraction***

Data extraction will be carried out by XYX and HK using a pro-forma to be developed by XYX for this purpose. These parameters in our study included author, publication year, region, method, patient sex, age, number, underlying chronic liver disease etiology, histological scoring system, average liver biopsy length, duration of time between biopsy and laboratory tests, prevalence of the fibrosis stage, as well as cutoff values to identify the fibrosis stage. [10,11]

***Quality Assessment***

Study quality will be appraised using the quality assessment of diagnostic accuracy studies (QUADAS) questionnaire [12] that contain a checklist of 14 items that should be included in cohort studies.

### How to assess Study quality by QUADAS?

#### 1. Was the spectrum of patients representative of the patients who will receive the test in practice?

Studies should score "yes" for this item if you believe, based on the information reported or obtained from the study's authors, that the spectrum of patients included in the study was representative of those in whom the test will be used in practice. The judgement should be based on both the method of recruitment and the characteristics of those recruited. Studies which recruit a group of healthy controls and a group known to have the target disorder will be coded as "no" on this item in nearly all circumstances. Reviewers should pre-specify in the protocol of the review what spectrum of patients would be acceptable taking factors such as disease prevalence and severity, age, and sex, into account. If you think that the population studied does not fit into what you specified as acceptable, the item should be scored as "no". If there is insufficient information available to make a judgement then it should be scored as "unclear".

#### 2. Were selection criteria clearly described?

If you think that all relevant information regarding how participants were selected for inclusion in the study has been provided then this item should be scored as "yes". If study selection criteria are not clearly reported then this item should be scored as "no". In situations where selection criteria are partially reported and you feel that you do not have enough information to score this item as "yes", then it should be scored as "unclear".

#### 3. Is the reference standard likely to correctly classify the target condition?

If you believe that the reference standard is likely to correctly classify the target condition or is the best method available, then this item should be scored "yes". Making a judgement as to the accuracy of the reference standard may not be straightforward. You may need experience of the topic area to know whether a test is an appropriate reference standard, or if a combination of tests are used you may have to consider carefully whether these were appropriate. If you do not think that the reference standard was likely to have correctly classified the target condition then this item should be scored as "no". If there is insufficient information to make a judgement then this should be scored as "unclear".

#### 4. Is the time period between reference standard and index test short enough to be reasonably sure that the target condition did not change between the two tests?

When to score this item as "yes" is related to the target condition. For conditions that progress rapidly even a delay of several days may be important. For such conditions this item should be scored "yes" if the delay between the performance of the index and reference standard is very short, a matter of hours or days. However, for chronic conditions disease status is unlikely to change in a week, or a month, or even longer. In such conditions longer delays between performance of the index and reference standard may be scored as "yes". You will have to make judgements regarding what is considered "short enough". You should think about this before starting work on a review, and define what you consider to be "short enough" for the specific topic area that you are reviewing. If you think the time period between the performance of the index test and the reference standard was sufficiently long that disease status may have changed between the performance of the two tests then this item should be scored as "no". If insufficient information is provided this should be scored as "unclear".

#### 5. Did the whole sample or a random selection of the sample, receive verification using a reference standard?

If it is clear from the study that all patients, or a random selection of patients, who received the index test went on to receive verification of their disease status using a reference standard then this item should be scored as "yes". This item should be scored as yes even if the reference standard was not the same for all patients. If some of the patients who received the index test did not receive verification of their true disease state, and the selection of patients to receive the reference standard was not random, then this item should be scored as "no". If this information is not reported by the study then it should be scored as "unclear".

#### 6. Did patients receive the same reference standard regardless of the index test result?

If it is clear that patients received verification of their true disease status using the same reference standard then this item should be scored as "yes". If some patients received verification using a different reference standard this item should be scored as "no". If this information is not reported by the study then it should be scored as "unclear".

#### 7. Was the reference standard independent of the index test (i.e. the index test did not form part of the reference standard)?

If it is clear from the study that the index test did not form part of the reference standard then this item should be scored as "yes". If it appears that the index test formed part of the reference standard then this item should be scored as "no". If this information is not reported by the study then it should be scored as "unclear".

#### 8. Was the execution of the index test described in sufficient detail to permit replication of the test?

#### 9. Was the execution of the reference standard described in sufficient detail to permit its replication?

If the study reports sufficient details or citations to permit replication of the index test and reference standard then these items should be scored as "yes". In other cases these items should be scored as "no". In situations where details of test performance are partially reported and you feel that you do not have enough information to score this item as "yes", then it should be scored as "unclear".

#### 10. Were the index test results interpreted without knowledge of the results of the reference standard?

#### 11. Were the reference standard results interpreted without knowledge of the results of the index test?

If the study clearly states that the test results (index or reference standard) were interpreted blind to the results of the other test then these items should be scored as "yes". If this does not appear to be the case they should be scored as "no". If this information is not reported by the study then it should be scored as "unclear".

#### 12. Were the same clinical data available when test results were interpreted as would be available when the test is used in practice?

If clinical data would normally be available when the test is interpreted in practice and similar data were available when interpreting the index test in the study then this item should be scored as "yes". Similarly, if clinical data would not be available in practice and these data were not available when the index test results were interpreted then this item should be scored as "yes". If this is not the case then this item should be scored as "no". If this information is not reported by the study then it should be scored as "unclear".

#### 13. Were uninterpretable/ intermediate test results reported?

If it is clear that all test results, including uninterpretable/indeterminate/intermediate are reported then this item should be scored as "yes". If you think that such results occurred but have not been reported then this item should be scored as "no". If it is not clear whether all study results have been reported then this item should be scored as "unclear".

#### 14. Were withdrawals from the study explained?

If it is clear what happened to all patients who entered the study, for example if a flow diagram of study participants is reported, then this item should be scored as "yes". If it appears that some of the participants who entered the study did not complete the study, i.e. did not receive both the index test and reference standard, and these patients were not accounted for then this item should be scored as "no". If it is not clear whether all patients who entered the study were accounted for then this item should be scored as "unclear".

***Statistical Analysis***

Meta-analysis will be considered if there are sufficient studies available with suitable data. If it is not possible to conduct a meta-analysis, due to the heterogeneity of the studies, a meta-regression was conducted to further explore the covariates that may induce the heterogeneity, according to the some predefined characteristics. In order to provide clinically meaningful results, three measures of diagnostic test accuracy were examined: Area under the SROC curve, Diagnostic Odds Ratio, and Summary Sensitivities and Specificities.

# *Reference*

1. Custer, B., et al., *Global epidemiology of hepatitis B virus.* J Clin Gastroenterol, 2004. **38**(10 Suppl 3): p. S158-68.

2. Gumusay, O., et al., *Diagnostic potential of serum direct markers and non-invasive fibrosis models in patients with chronic hepatitis B.* Hepatol Res, 2013. **43**(3): p. 228-37.

3. Liaw, Y.F., et al., *Lamivudine for patients with chronic hepatitis B and advanced liver disease.* N Engl J Med, 2004. **351**(15): p. 1521-31.

4. Bravo, A.A., S.G. Sheth, and S. Chopra, *Liver biopsy.* N Engl J Med, 2001. **344**(7): p. 495-500.

5. Colloredo, G., et al., *Impact of liver biopsy size on histological evaluation of chronic viral hepatitis: the smaller the sample, the milder the disease.* J Hepatol, 2003. **39**(2): p. 239-44.

6. Kim, B.K., et al., *Validation of FIB-4 and comparison with other simple noninvasive indices for predicting liver fibrosis and cirrhosis in hepatitis B virus-infected patients.* Liver Int, 2010. **30**(4): p. 546-53.

7. Sterling, R.K., et al., *Similar progression of fibrosis between HIV/HCV-infected and HCV-infected patients: Analysis of paired liver biopsy samples.* Clin Gastroenterol Hepatol, 2010. **8**(12): p. 1070-6.

8. Deeks, J.J., *Systematic reviews in health care: Systematic reviews of evaluations of diagnostic and screening tests.* BMJ, 2001. **323**(7305): p. 157-62.

9. Chrysanthos, N.V., et al., *Aspartate aminotransferase to platelet ratio index for fibrosis evaluation in chronic viral hepatitis.* Eur J Gastroenterol Hepatol, 2006. **18**(4): p. 389-96.

10. Wang, H., et al., *Comparison of FIB-4 and APRI in Chinese HBV-infected patients with persistently normal ALT and mildly elevated ALT.* J Viral Hepat, 2013. **20**(4): p. e3-10.

11. Lin, Z.H., et al., *Performance of the aspartate aminotransferase-to-platelet ratio index for the staging of hepatitis C-related fibrosis: an updated meta-analysis.* Hepatology, 2011. **53**(3): p. 726-36.

12. Whiting, P., et al., *The development of QUADAS: a tool for the quality assessment of studies of diagnostic accuracy included in systematic reviews.* BMC Med Res Methodol, 2003. **3**: p. 25.
