# Supplementary material for: The Effectiveness of Noninvasive Biomarkers to Predict Hepatitis B-Related Significant Fibrosis and Cirrhosis: A Systematic Review and Meta-Analysis of Diagnostic Test Accuracy
Source: PLoS One. 2014 Jun 25;9(6):e100182. doi: 10.1371/journal.pone.0100182 (PMC4070977; doi:10.1371/journal.pone.0100182)
Supplement: Text S2 — Search strategies. (DOC) [file pone.0100182.s011.doc]

***Text S3***

***Search strategies***

Medline search strategy

We used the following search terms to search all databases: HBV; CHB; hepatitis B virus; chronic hepatitis B; APRI; aspartate aminotransferase-to-platelet ratio index; FIB-4; fibrosis index based on the 4 factors; FibroTest

Search strategy: MEDLINE

1. CHB/
2. CHB.tw
3. chronic hepatitis B/
4. chronic hepatitis B.tw
5. 01 or 02 or 03 or 04
6. HBV/
7. HBV.tw
8. hepatitis B virus/
9. hepatitis B virus.tw
10. 06 or 07 or 08 or 09
11. 05 or 10
12. APRI/
13. APRI.tw
14. aspartate aminotransferase-to-platelet ratio index/
15. aspartate aminotransferase-to-platelet ratio index.tw
16. FIB-4/
17. FIB-4.tw
18. fibrosis index based on the 4 factors/
19. fibrosis index based on the 4 factors.tw
20. FibroTest/
21. FibroTest.tw
22. 12 or 13 or 14 or 15 or 16 or 17 or 18 or 19 or 20 or 21
23. 11 and 22
