# Supplementary material for: The Effectiveness of Noninvasive Biomarkers to Predict Hepatitis B-Related Significant Fibrosis and Cirrhosis: A Systematic Review and Meta-Analysis of Diagnostic Test Accuracy
Source: PLoS One. 2014 Jun 25;9(6):e100182. doi: 10.1371/journal.pone.0100182 (PMC4070977; doi:10.1371/journal.pone.0100182)
Supplement: Text S3 — Quadas checklist. (DOC) [file pone.0100182.s012.doc]

Text S3

Quadas checklist

**QUADAS checklist**. The quality assessment of studies of diagnostic accuracy checklist with description of checklist items.

|  | **QUADAS Item** | **Code 1 2 3 4** |
| --- | --- | --- |
| Was the spectrum of patients representative of the patients who will receive the test in practice? | **1** | Yes □ No □ Unclear □ |
| Were selection criteria clearly described? | **2** | Yes □ No □ Unclear □ |
| Is the reference standard likely to correctly classify the target condition? | **3** | Yes □ No □ Unclear □ |
| Is the time period between reference standard and index test short enough to be reasonably sure that the target condition did not change between the two tests? | **4** | Yes □ No □ Unclear □ |
| Did the whole study population or a random selection of the sample, receive verification using a reference standard for diagnosis? | **5** | Yes □ No □ Unclear □ |
| Did patients receive the same reference standard regardless of the index test result? | **6** | Yes □ No □ Unclear □ |
| Was the reference standard independent of the index test? | **7** | Yes □ No □ Unclear □ |
| Was the execution of the index test described in sufficient detail to permit replication of the test? | **8** | Yes □ No □ Unclear □ |
| Was the execution of the reference standard described in sufficient detail to permit its replication? | **9** | Yes □ No □ Unclear □ |
| Were the index test results interpreted without the knowledge of the results of the reference standard? | **10** | Yes □ No □ Unclear □ |
| Were the reference standard results interpreted without knowledge of the index test results? | **11** | Yes □ No □ Unclear □ |
| Were the same clinical data available when test results were interpreted as would be available when the test is used in practice? | **12** | Yes □ No □ Unclear □ |
| Were uninterpretable / intermediate test results reported? | **13** | Yes □ No □ Unclear □ |
| Were withdrawals from the study explained? | **14** | Yes □ No □ Unclear □ |

##### Users' guide to QUADAS

##### How to score this item

#### 1. Was the spectrum of patients representative of the patients who will receive the test in practice?

Studies should score "yes" for this item if you believe, based on the information reported or obtained from the study's authors, that the spectrum of patients included in the study was representative of those in whom the test will be used in practice. The judgement should be based on both the method of recruitment and the characteristics of those recruited. Studies which recruit a group of healthy controls and a group known to have the target disorder will be coded as "no" on this item in nearly all circumstances. Reviewers should pre-specify in the protocol of the review what spectrum of patients would be acceptable taking factors such as disease prevalence and severity, age, and sex, into account. If you think that the population studied does not fit into what you specified as acceptable, the item should be scored as "no". If there is insufficient information available to make a judgement then it should be scored as "unclear".

#### 2. Were selection criteria clearly described?

If you think that all relevant information regarding how participants were selected for inclusion in the study has been provided then this item should be scored as "yes". If study selection criteria are not clearly reported then this item should be scored as "no". In situations where selection criteria are partially reported and you feel that you do not have enough information to score this item as "yes", then it should be scored as "unclear".

#### 3. Is the reference standard likely to correctly classify the target condition?

If you believe that the reference standard is likely to correctly classify the target condition or is the best method available, then this item should be scored "yes". Making a judgement as to the accuracy of the reference standard may not be straightforward. You may need experience of the topic area to know whether a test is an appropriate reference standard, or if a combination of tests are used you may have to consider carefully whether these were appropriate. If you do not think that the reference standard was likely to have correctly classified the target condition then this item should be scored as "no". If there is insufficient information to make a judgement then this should be scored as "unclear".

#### 4. Is the time period between reference standard and index test short enough to be reasonably sure that the target condition did not change between the two tests?

When to score this item as "yes" is related to the target condition. For conditions that progress rapidly even a delay of several days may be important. For such conditions this item should be scored "yes" if the delay between the performance of the index and reference standard is very short, a matter of hours or days. However, for chronic conditions disease status is unlikely to change in a week, or a month, or even longer. In such conditions longer delays between performance of the index and reference standard may be scored as "yes". You will have to make judgements regarding what is considered "short enough". You should think about this before starting work on a review, and define what you consider to be "short enough" for the specific topic area that you are reviewing. If you think the time period between the performance of the index test and the reference standard was sufficiently long that disease status may have changed between the performance of the two tests then this item should be scored as "no". If insufficient information is provided this should be scored as "unclear".

#### 5. Did the whole sample or a random selection of the sample, receive verification using a reference standard?

If it is clear from the study that all patients, or a random selection of patients, who received the index test went on to receive verification of their disease status using a reference standard then this item should be scored as "yes". This item should be scored as yes even if the reference standard was not the same for all patients. If some of the patients who received the index test did not receive verification of their true disease state, and the selection of patients to receive the reference standard was not random, then this item should be scored as "no". If this information is not reported by the study then it should be scored as "unclear".

#### 6. Did patients receive the same reference standard regardless of the index test result?

If it is clear that patients received verification of their true disease status using the same reference standard then this item should be scored as "yes". If some patients received verification using a different reference standard this item should be scored as "no". If this information is not reported by the study then it should be scored as "unclear".

#### 7. Was the reference standard independent of the index test (i.e. the index test did not form part of the reference standard)?

If it is clear from the study that the index test did not form part of the reference standard then this item should be scored as "yes". If it appears that the index test formed part of the reference standard then this item should be scored as "no". If this information is not reported by the study then it should be scored as "unclear".

#### 8. Was the execution of the index test described in sufficient detail to permit replication of the test?

#### 9. Was the execution of the reference standard described in sufficient detail to permit its replication?

If the study reports sufficient details or citations to permit replication of the index test and reference standard then these items should be scored as "yes". In other cases these items should be scored as "no". In situations where details of test performance are partially reported and you feel that you do not have enough information to score this item as "yes", then it should be scored as "unclear".

#### 10. Were the index test results interpreted without knowledge of the results of the reference standard?

#### 11. Were the reference standard results interpreted without knowledge of the results of the index test?

If the study clearly states that the test results (index or reference standard) were interpreted blind to the results of the other test then these items should be scored as "yes". If this does not appear to be the case they should be scored as "no". If this information is not reported by the study then it should be scored as "unclear".

#### 12. Were the same clinical data available when test results were interpreted as would be available when the test is used in practice?

If clinical data would normally be available when the test is interpreted in practice and similar data were available when interpreting the index test in the study then this item should be scored as "yes". Similarly, if clinical data would not be available in practice and these data were not available when the index test results were interpreted then this item should be scored as "yes". If this is not the case then this item should be scored as "no". If this information is not reported by the study then it should be scored as "unclear".

#### 13. Were uninterpretable/ intermediate test results reported?

If it is clear that all test results, including uninterpretable/indeterminate/intermediate are reported then this item should be scored as "yes". If you think that such results occurred but have not been reported then this item should be scored as "no". If it is not clear whether all study results have been reported then this item should be scored as "unclear".

#### 14. Were withdrawals from the study explained?

If it is clear what happened to all patients who entered the study, for example if a flow diagram of study participants is reported, then this item should be scored as "yes". If it appears that some of the participants who entered the study did not complete the study, i.e. did not receive both the index test and reference standard, and these patients were not accounted for then this item should be scored as "no". If it is not clear whether all patients who entered the study were accounted for then this item should be scored as "unclear".

The majority of items included in QUADAS relate to bias (items 3, 4, 5, 6, 7, 10, 11, 12 and 14), with only two items each relating to variability (items 1 and 2) and reporting (items 8, 9 and 13).
