# Supplementary material for: The Effectiveness of Noninvasive Biomarkers to Predict Hepatitis B-Related Significant Fibrosis and Cirrhosis: A Systematic Review and Meta-Analysis of Diagnostic Test Accuracy
Source: PLoS One. 2014 Jun 25;9(6):e100182. doi: 10.1371/journal.pone.0100182 (PMC4070977; doi:10.1371/journal.pone.0100182)
Supplement: Text S4 — Meta-regression of APRI detecting significant fibrosis. (DOC) [file pone.0100182.s013.doc]

Text S5  Meta-regression analysis of the APRI for detecting significant fibrosis 


1 step
-----------------------------------------------------------------------------------------
Meta-Regression(Inverse Variance weights) 

 Var		  Coeff.	Std. Err.	p - value	    RDOR 	    [95%CI]
-----------------------------------------------------------------------------------------
Cte.         	  10.646	  4.8001	  0.0773	    ----  	     ----  
S            	  -0.163	  0.1498	  0.3261	    ----  	     ----  
location    	  -0.186	  0.6644	  0.7909	    0.83	  (0.15;4.58)
samplesize  	   0.000	  0.0015	  0.8502	    1.00	  (1.00;1.00)
MedianAge   	  -0.292	  0.4451	  0.5410	    0.75	  (0.24;2.34)
males       	   0.507	  2.4792	  0.8461	    1.66	  (0.00;972.15)
Etiology    	   0.449	  0.7241	  0.5620	    1.57	  (0.24;10.08)
LBSystem    	   0.675	  0.3987	  0.1513	    1.96	  (0.70;5.47)
LBLength    	  -0.027	  0.2666	  0.9234	    0.97	  (0.49;1.93)
Prevalence  	  -1.582	  2.0735	  0.4798	    0.21	  (0.00;42.41)
QUADAS      	  -0.554	  0.3011	  0.1252	    0.57	  (0.27;1.25)
Design      	  -1.256	  0.9062	  0.2244	    0.28	  (0.03;2.93)

-----------------------------------------------------------------------------------------

2 step
-----------------------------------------------------------------------------------------
Meta-Regression(Inverse Variance weights) 

 Var		  Coeff.	Std. Err.	p - value	    RDOR 	    [95%CI]
-----------------------------------------------------------------------------------------
Cte.         	  10.459	  4.1922	  0.0469	    ----  	     ----  
S            	  -0.164	  0.1315	  0.2578	    ----  	     ----  
location    	  -0.192	  0.5574	  0.7425	    0.83	  (0.21;3.23)
samplesize  	   0.000	  0.0013	  0.8696	    1.00	  (1.00;1.00)
MedianAge   	  -0.310	  0.3863	  0.4529	    0.73	  (0.29;1.89)
males       	   0.429	  2.0172	  0.8386	    1.54	  (0.01;213.78)
Etiology    	   0.477	  0.6073	  0.4621	    1.61	  (0.36;7.12)
LBSystem    	   0.654	  0.3492	  0.1104	    1.92	  (0.82;4.52)
Prevalence  	  -1.625	  1.8353	  0.4100	    0.20	  (0.00;17.56)
QUADAS      	  -0.543	  0.2630	  0.0845	    0.58	  (0.31;1.11)
Design      	  -1.171	  0.7187	  0.1543	    0.31	  (0.05;1.80)

-----------------------------------------------------------------------------------------

3 step
-----------------------------------------------------------------------------------------
Meta-Regression(Inverse Variance weights) 

 Var		  Coeff.	Std. Err.	p - value	    RDOR 	    [95%CI]
-----------------------------------------------------------------------------------------
Cte.         	  10.494	  3.5158	  0.0204	    ----  	     ----  
S            	  -0.155	  0.1123	  0.2108	    ----  	     ----  
location    	  -0.167	  0.4992	  0.7483	    0.85	  (0.26;2.76)
samplesize  	   0.000	  0.0011	  0.9561	    1.00	  (1.00;1.00)
MedianAge   	  -0.379	  0.2582	  0.1854	    0.68	  (0.37;1.26)
Etiology    	   0.552	  0.4689	  0.2779	    1.74	  (0.57;5.26)
LBSystem    	   0.618	  0.3033	  0.0810	    1.86	  (0.91;3.80)
Prevalence  	  -1.504	  1.5402	  0.3612	    0.22	  (0.01;8.48)
QUADAS      	  -0.525	  0.2332	  0.0591	    0.59	  (0.34;1.03)
Design      	  -1.109	  0.6326	  0.1231	    0.33	  (0.07;1.47)

-----------------------------------------------------------------------------------------

4 step
-----------------------------------------------------------------------------------------
Meta-Regression(Inverse Variance weights) 

 Var		  Coeff.	Std. Err.	p - value	    RDOR 	    [95%CI]
-----------------------------------------------------------------------------------------
Cte.         	  10.187	  3.0343	  0.0100	    ----  	     ----  
S            	  -0.155	  0.0983	  0.1539	    ----  	     ----  
location    	  -0.137	  0.4417	  0.7639	    0.87	  (0.31;2.41)
MedianAge   	  -0.398	  0.2141	  0.0999	    0.67	  (0.41;1.10)
Etiology    	   0.545	  0.4001	  0.2104	    1.72	  (0.69;4.34)
LBSystem    	   0.583	  0.2603	  0.0555	    1.79	  (0.98;3.27)
Prevalence  	  -1.462	  1.2392	  0.2720	    0.23	  (0.01;4.04)
QUADAS      	  -0.503	  0.1991	  0.0354	    0.60	  (0.38;0.96)
Design      	  -1.052	  0.5291	  0.0820	    0.35	  (0.10;1.18)

-----------------------------------------------------------------------------------------


5 step
-----------------------------------------------------------------------------------------
Meta-Regression(Inverse Variance weights) 

 Var		  Coeff.	Std. Err.	p - value	    RDOR 	    [95%CI]
-----------------------------------------------------------------------------------------
Cte.         	   9.808	  2.5699	  0.0041	    ----  	     ----  
S            	  -0.162	  0.0846	  0.0885	    ----  	     ----  
MedianAge   	  -0.444	  0.1696	  0.0280	    0.64	  (0.44;0.94)
Etiology    	   0.480	  0.3396	  0.1916	    1.62	  (0.75;3.48)
LBSystem    	   0.512	  0.2062	  0.0348	    1.67	  (1.05;2.66)
Prevalence  	  -1.306	  0.9559	  0.2052	    0.27	  (0.03;2.36)
QUADAS      	  -0.475	  0.1694	  0.0205	    0.62	  (0.42;0.91)
Design      	  -0.997	  0.4494	  0.0537	    0.37	  (0.13;1.02)

-----------------------------------------------------------------------------------------

6 step
-----------------------------------------------------------------------------------------
Meta-Regression(Inverse Variance weights) 

 Var		  Coeff.	Std. Err.	p - value	    RDOR 	    [95%CI]
-----------------------------------------------------------------------------------------
Cte.         	   9.646	  2.5671	  0.0037	    ----  	     ----  
S            	  -0.196	  0.0808	  0.0359	    ----  	     ----  
MedianAge   	  -0.547	  0.1517	  0.0048	    0.58	  (0.41;0.81)
Etiology    	   0.304	  0.3145	  0.3558	    1.36	  (0.67;2.73)
LBSystem    	   0.375	  0.1800	  0.0640	    1.45	  (0.97;2.17)
QUADAS      	  -0.442	  0.1677	  0.0249	    0.64	  (0.44;0.93)
Design      	  -1.042	  0.4481	  0.0424	    0.35	  (0.13;0.96)

-----------------------------------------------------------------------------------------

7 step
-----------------------------------------------------------------------------------------
Meta-Regression(Inverse Variance weights) 

 Var		  Coeff.	Std. Err.	p - value	    RDOR 	    [95%CI]
-----------------------------------------------------------------------------------------
Cte.         	  10.194	  2.5039	  0.0018	    ----  	     ----  
S            	  -0.228	  0.0738	  0.0103	    ----  	     ----  
MedianAge   	  -0.486	  0.1380	  0.0048	    0.61	  (0.45;0.83)
LBSystem    	   0.318	  0.1701	  0.0886	    1.37	  (0.94;2.00)
QUADAS      	  -0.469	  0.1653	  0.0161	    0.63	  (0.43;0.90)
Design      	  -0.950	  0.4379	  0.0529	    0.39	  (0.15;1.01)

-----------------------------------------------------------------------------------------


8 step
-----------------------------------------------------------------------------------------
Meta-Regression(Inverse Variance weights) 

 Var		  Coeff.	Std. Err.	p - value	    RDOR 	    [95%CI]
-----------------------------------------------------------------------------------------
Cte.         	   7.877	  2.1751	  0.0035	    ----  	     ----  
S            	  -0.231	  0.0738	  0.0086	    ----  	     ----  
MedianAge   	  -0.455	  0.1370	  0.0061	    0.63	  (0.47;0.86)
QUADAS      	  -0.300	  0.1383	  0.0507	    0.74	  (0.55;1.00)
Design      	  -0.574	  0.3889	  0.1658	    0.56	  (0.24;1.31)

-----------------------------------------------------------------------------------------


9 step
-----------------------------------------------------------------------------------------
Meta-Regression(Inverse Variance weights) 

 Var		  Coeff.	Std. Err.	p - value	    RDOR 	    [95%CI]
-----------------------------------------------------------------------------------------
Cte.         	   6.709	  2.0261	  0.0056	    ----  	     ----  
S            	  -0.207	  0.0719	  0.0129	    ----  	     ----  
MedianAge   	  -0.455	  0.1370	  0.0056	    0.63	  (0.47;0.85)
QUADAS      	  -0.297	  0.1383	  0.0513	    0.74	  (0.55;1.00)

-----------------------------------------------------------------------------------------


10 step
-----------------------------------------------------------------------------------------
Meta-Regression(Inverse Variance weights) 

 Var		  Coeff.	Std. Err.	p - value	    RDOR 	    [95%CI]
-----------------------------------------------------------------------------------------
Cte.         	   2.401	  0.2782	  0.0000	    ----  	     ----  
S            	  -0.129	  0.0620	  0.0564	    ----  	     ----  
MedianAge   	  -0.309	  0.1191	  0.0211	    0.73	  (0.57;0.95)

-----------------------------------------------------------------------------------------


11 step
-----------------------------------------------------------------------------------------
Meta-Regression(Inverse Variance weights) 

 Var		  Coeff.	Std. Err.	p - value	    RDOR 	    [95%CI]
-----------------------------------------------------------------------------------------
Cte.         	   4.258	  2.6374	  0.1288	    ----  	     ----  
S            	  -0.163	  0.0971	  0.1150	    ----  	     ----  
QUADAS      	  -0.189	  0.1990	  0.3579	    0.83	  (0.54;1.27)

-----------------------------------------------------------------------------------------
