# Supplementary material for: The Effectiveness of Noninvasive Biomarkers to Predict Hepatitis B-Related Significant Fibrosis and Cirrhosis: A Systematic Review and Meta-Analysis of Diagnostic Test Accuracy
Source: PLoS One. 2014 Jun 25;9(6):e100182. doi: 10.1371/journal.pone.0100182 (PMC4070977; doi:10.1371/journal.pone.0100182)
Supplement: Text S5 — Meta-regression of FibroTest detecting significant fibrosis. (RTF) [file pone.0100182.s014.rtf]

Text S6  Meta-regression analysis of the FibroTest for detecting significant fibrosis


1 step
-----------------------------------------------------------------------------------------
Meta-Regression(Inverse Variance weights) 

 Var		  Coeff.	Std. Err.	p - value	    RDOR 	    [95%CI]
-----------------------------------------------------------------------------------------
Cte.         	   2.986	  8.4288	  0.7832	    ----  	     ----  
S            	   0.153	  0.5834	  0.8370	    ----  	     ----  
location  	   1.858	  4.8786	  0.7683	    6.41	  (0.00;5345979963684500000000000000.00)
samplesize        -1.480	  1.0905	  0.4042	    0.23	  (0.00;237081.00)
MedianAge     	   0.239	  0.2127	  0.4627	    1.27	  (0.09;18.95)
males         	  -4.004	 10.8959	  0.7758	    0.02	  (0.00;24393130190728000000000000000000000000000000000000000000000.00)
Etiology    	  -1.789	  1.0620	  0.3410	    0.17	  (0.00;121127.20)
LBSystem    	  -1.251	  3.1494	  0.7593	    0.29	  (0.00;68516433887622500.00)
LBLength    	  -0.805	  0.9072	  0.5381	    0.45	  (0.00;45383.15)
Prevalence  	  -1.605	  4.8048	  0.7948	    0.20	  (0.00;65638196194002500000000000.00)

-----------------------------------------------------------------------------------------

2 step
-----------------------------------------------------------------------------------------
Meta-Regression(Inverse Variance weights) 

 Var		  Coeff.	Std. Err.	p - value	    RDOR 	    [95%CI]
-----------------------------------------------------------------------------------------
Cte.         	   1.993	  8.1651	  0.8476	    ----  	     ----  
S            	   0.417	  0.4252	  0.5063	    ----  	     ----  
location  	   1.672	  4.2821	  0.7630	    5.32	  (0.00;2266891330868930000000000.00)
samplesize        -1.752	  0.8161	  0.2775	    0.17	  (0.00;5526.78)
MeanAge     	   0.304	  0.2180	  0.3959	    1.36	  (0.08;21.64)
males        	  -5.219	  9.1701	  0.6706	    0.01	  (0.00;2169083223211590000000000000000000000000000000000.00)
Etiology    	  -2.587	  1.9914	  0.4176	    0.08	  (0.00;7339828807.02)
LBSystem    	  -0.941	  2.6674	  0.7840	    0.39	  (0.00;204475470161160.00)
LBLength    	  -1.361	  0.9658	  0.3929	    0.26	  (0.00;54747.81)
Design         	   0.664	  1.4524	  0.7271	    1.94	  (0.00;200949976.73)

-----------------------------------------------------------------------------------------

3 step
-----------------------------------------------------------------------------------------
Meta-Regression(Inverse Variance weights) 

 Var		  Coeff.	Std. Err.	p - value	    RDOR 	    [95%CI]
-----------------------------------------------------------------------------------------
Cte.         	   1.973	  6.7159	  0.7966	    ----  	     ----  
S            	   0.521	  0.3017	  0.2261	    ----  	     ----  
location  	   0.204	  0.6036	  0.7680	    1.23	  (0.09;16.45)
samplesize    	  -1.791	  0.6061	  0.0980	    0.17	  (0.01;2.26)
MeanAge     	   0.365	  0.1479	  0.1326	    1.44	  (0.76;2.72)
males         	  -7.478	  5.8586	  0.3300	    0.00	  (0.00;50093263.11)
Etiology    	  -2.754	  1.6387	  0.2348	    0.06	  (0.00;73.46)
LBLength    	  -1.354	  0.7562	  0.2152	    0.26	  (0.01;6.68)
Design         	   0.705	  1.2217	  0.6222	    2.02	  (0.01;388.23)

-----------------------------------------------------------------------------------------


4 step
-----------------------------------------------------------------------------------------
Meta-Regression(Inverse Variance weights) 

 Var		  Coeff.	Std. Err.	p - value	    RDOR 	    [95%CI]
-----------------------------------------------------------------------------------------
Cte.         	   2.562	  6.4853	  0.7193	    ----  	     ----  
S            	   0.490	  0.2872	  0.1864	    ----  	     ----  
samplesize     	  -1.743	  0.5891	  0.0596	    0.18	  (0.03;1.14)
MeanAge     	   0.349	  0.1407	  0.0891	    1.42	  (0.91;2.22)
males         	  -7.424	  5.8564	  0.2943	    0.00	  (0.00;74117.15)
Etiology    	  -2.616	  1.5868	  0.1978	    0.07	  (0.00;11.40)
LBLength    	  -1.232	  0.6646	  0.1607	    0.29	  (0.04;2.42)
Design         	   0.506	  1.0702	  0.6685	    1.66	  (0.06;50.00)

-----------------------------------------------------------------------------------------

5 step
-----------------------------------------------------------------------------------------
Meta-Regression(Inverse Variance weights) 

 Var		  Coeff.	Std. Err.	p - value	    RDOR 	    [95%CI]
-----------------------------------------------------------------------------------------
Cte.         	   3.499	  6.1751	  0.6012	    ----  	     ----  
S            	   0.423	  0.2496	  0.1653	    ----  	     ----  
samplesize    	  -1.749	  0.5889	  0.0412	    0.17	  (0.03;0.89)
MeanAge     	   0.311	  0.1155	  0.0544	    1.37	  (0.99;1.88)
males         	  -7.680	  5.8314	  0.2582	    0.00	  (0.00;4961.89)
Etiology    	  -1.933	  0.6576	  0.0424	    0.14	  (0.02;0.90)
LBLength    	  -0.988	  0.4177	  0.0772	    0.37	  (0.12;1.19)

-----------------------------------------------------------------------------------------

6 step
-----------------------------------------------------------------------------------------
Meta-Regression(Inverse Variance weights) 

 Var		  Coeff.	Std. Err.	p - value	    RDOR 	    [95%CI]
-----------------------------------------------------------------------------------------
Cte.         	  -3.452	  3.2058	  0.3307	    ----  	     ----  
S            	   0.423	  0.2496	  0.1510	    ----  	     ----  
samplesize    	  -1.199	  0.4158	  0.0344	    0.30	  (0.10;0.88)
MeanAge     	   0.301	  0.1152	  0.0474	    1.35	  (1.01;1.82)
Etiology    	  -1.849	  0.6545	  0.0369	    0.16	  (0.03;0.85)
LBLength    	  -0.618	  0.3091	  0.1020	    0.54	  (0.24;1.19)

-----------------------------------------------------------------------------------------

7 step
-----------------------------------------------------------------------------------------
Meta-Regression(Inverse Variance weights) 

 Var		  Coeff.	Std. Err.	p - value	    RDOR 	    [95%CI]
-----------------------------------------------------------------------------------------
Cte.         	  -1.135	  3.4398	  0.7527	    ----  	     ----  
S            	   0.106	  0.2585	  0.6953	    ----  	     ----  
samplesize    	  -0.574	  0.3431	  0.1456	    0.56	  (0.24;1.30)
MeanAge     	   0.147	  0.0953	  0.1747	    1.16	  (0.92;1.46)
Etiology    	  -0.785	  0.4112	  0.1049	    0.46	  (0.17;1.25)

-----------------------------------------------------------------------------------------

8 step
-----------------------------------------------------------------------------------------
Meta-Regression(Inverse Variance weights) 

 Var		  Coeff.	Std. Err.	p - value	    RDOR 	    [95%CI]
-----------------------------------------------------------------------------------------
Cte.         	   4.055	  0.7101	  0.0007	    ----  	     ----  
S            	  -0.203	  0.1860	  0.3118	    ----  	     ----  
samplesize 	  -0.199	  0.2629	  0.4730	    0.82	  (0.44;1.53)
Etiology    	  -0.774	  0.4391	  0.1215	    0.46	  (0.16;1.30)

-----------------------------------------------------------------------------------------

9 step
-----------------------------------------------------------------------------------------
Meta-Regression(Inverse Variance weights) 

 Var		  Coeff.	Std. Err.	p - value	    RDOR 	    [95%CI]
-----------------------------------------------------------------------------------------
Cte.         	   3.809	  0.6360	  0.0003	    ----  	     ----  
S            	  -0.196	  0.1872	  0.3246	    ----  	     ----  
Etiology    	  -0.898	  0.4108	  0.0603	    0.41	  (0.16;1.05)

-----------------------------------------------------------------------------------------

10 step
-----------------------------------------------------------------------------------------
Meta-Regression(Inverse Variance weights) 

 Var		  Coeff.	Std. Err.	p - value	    RDOR 	    [95%CI]
-----------------------------------------------------------------------------------------
Cte.         	   3.283	  0.6454	  0.0009	    ----  	     ----  
S            	  -0.294	  0.2160	  0.2105	    ----  	     ----  
samplesize	  -0.354	  0.2867	  0.2516	    0.70	  (0.36;1.36)

-----------------------------------------------------------------------------------------

11 step
-----------------------------------------------------------------------------------------
Meta-Regression(Inverse Variance weights) 

 Var		  Coeff.	Std. Err.	p - value	    RDOR 	    [95%CI]
-----------------------------------------------------------------------------------------
Cte.         	   4.148	  0.6025	  0.0001	    ----  	     ----  
S            	  -0.384	  0.1780	  0.0629	    ----  	     ----  
center          	  -1.146	  0.3880	  0.0183	    0.32	  (0.13;0.78)

-----------------------------------------------------------------------------------------
