# Supplementary material for: The Effectiveness of Noninvasive Biomarkers to Predict Hepatitis B-Related Significant Fibrosis and Cirrhosis: A Systematic Review and Meta-Analysis of Diagnostic Test Accuracy
Source: PLoS One. 2014 Jun 25;9(6):e100182. doi: 10.1371/journal.pone.0100182 (PMC4070977; doi:10.1371/journal.pone.0100182)
Supplement: Text S6 — Meta-regression of APRI detecting cirrhosis. (RTF) [file pone.0100182.s015.rtf]

Text S7  Meta-regression analysis of the APRI for detecting cirrhosis


1 step
-----------------------------------------------------------------------------------------
Meta-Regression(Inverse Variance weights) 

 Var		  Coeff.	Std. Err.	p - value	    RDOR 	    [95%CI]
-----------------------------------------------------------------------------------------
Cte.         	   1.856	  1.3346	  0.3969	    ----  	     ----  
S            	  -0.128	  0.3222	  0.7595	    ----  	     ----  
location    	  -0.654	  1.5455	  0.7452	    0.52	  (0.00;175637739.14)
samplesize  	   0.008	  0.0164	  0.7237	    1.01	  (0.82;1.24)
MedianAge   	   1.559	  0.8741	  0.3253	    4.76	  (0.00;316864.88)
males       	  -0.255	  4.6868	  0.9654	    0.77	  (0.00;56541019990450300000000000.00)
Etiology    	  -2.472	  2.2660	  0.4723	    0.08	  (0.00;269456425718.20)
LBSystem    	  -0.036	  0.2132	  0.8944	    0.96	  (0.06;14.49)
LBLength    	   0.442	  1.2534	  0.7841	    1.56	  (0.00;12835942.84)
prevalence  	 -11.363	  9.8397	  0.4544	    0.00	  (0.00;23078618404098200000000000000000000000000000000000.00)

-----------------------------------------------------------------------------------------

2 step
-----------------------------------------------------------------------------------------
Meta-Regression(Inverse Variance weights) 

 Var		  Coeff.	Std. Err.	p - value	    RDOR 	    [95%CI]
-----------------------------------------------------------------------------------------
Cte.         	   1.851	  1.3315	  0.2990	    ----  	     ----  
S            	  -0.129	  0.3216	  0.7272	    ----  	     ----  
location    	  -0.659	  1.5424	  0.7107	    0.52	  (0.00;394.42)
samplesize  	   0.007	  0.0069	  0.4305	    1.01	  (0.98;1.04)
MedianAge   	   1.524	  0.5976	  0.1254	    4.59	  (0.35;60.10)
Etiology    	  -2.369	  1.2317	  0.1944	    0.09	  (0.00;18.74)
LBSystem    	  -0.043	  0.1716	  0.8271	    0.96	  (0.46;2.01)
LBLength    	   0.378	  0.4090	  0.4531	    1.46	  (0.25;8.48)
prevalence  	 -11.101	  8.5824	  0.3251	    0.00	  (0.00;164534728917.63)

-----------------------------------------------------------------------------------------

3 step
-----------------------------------------------------------------------------------------
Meta-Regression(Inverse Variance weights) 

 Var		  Coeff.	Std. Err.	p - value	    RDOR 	    [95%CI]
-----------------------------------------------------------------------------------------
Cte.         	   1.858	  1.3312	  0.2571	    ----  	     ----  
S            	  -0.152	  0.3080	  0.6557	    ----  	     ----  
location    	  -0.726	  1.5191	  0.6656	    0.48	  (0.00;60.87)
samplesize  	   0.006	  0.0066	  0.4125	    1.01	  (0.99;1.03)
MedianAge   	   1.497	  0.5870	  0.0840	    4.47	  (0.69;28.92)
Etiology    	  -2.312	  1.2099	  0.1520	    0.10	  (0.00;4.66)
LBLength    	   0.392	  0.4047	  0.4036	    1.48	  (0.41;5.37)
prevalence  	 -10.853	  8.5240	  0.2927	    0.00	  (0.00;11694813.08)

-----------------------------------------------------------------------------------------

4 step
-----------------------------------------------------------------------------------------
Meta-Regression(Inverse Variance weights) 

 Var		  Coeff.	Std. Err.	p - value	    RDOR 	    [95%CI]
-----------------------------------------------------------------------------------------
Cte.         	   1.529	  1.1392	  0.2506	    ----  	     ----  
S            	  -0.028	  0.1667	  0.8738	    ----  	     ----  
samplesize  	   0.008	  0.0050	  0.1689	    1.01	  (0.99;1.02)
MedianAge   	   1.572	  0.5653	  0.0498	    4.82	  (1.00;23.14)
Etiology    	  -2.689	  0.9163	  0.0426	    0.07	  (0.01;0.87)
LBLength    	   0.350	  0.3948	  0.4253	    1.42	  (0.47;4.25)
prevalence  	 -14.273	  4.6240	  0.0367	    0.00	  (0.00;0.24)

-----------------------------------------------------------------------------------------

5 step
-----------------------------------------------------------------------------------------
Meta-Regression(Inverse Variance weights) 

 Var		  Coeff.	Std. Err.	p - value	    RDOR 	    [95%CI]
-----------------------------------------------------------------------------------------
Cte.         	   2.324	  0.7031	  0.0214	    ----  	     ----  
S            	  -0.028	  0.1667	  0.8723	    ----  	     ----  
samplesize  	   0.004	  0.0021	  0.0920	    1.00	  (1.00;1.01)
MedianAge   	   1.260	  0.4425	  0.0359	    3.53	  (1.13;11.00)
Etiology    	  -2.005	  0.4947	  0.0098	    0.13	  (0.04;0.48)
prevalence  	 -12.072	  3.9011	  0.0270	    0.00	  (0.00;0.13)

-----------------------------------------------------------------------------------------

6 step
-----------------------------------------------------------------------------------------
Meta-Regression(Inverse Variance weights) 

 Var		  Coeff.	Std. Err.	p - value	    RDOR 	    [95%CI]
-----------------------------------------------------------------------------------------
Cte.         	   3.123	  0.5892	  0.0018	    ----  	     ----  
S            	  -0.114	  0.1615	  0.5055	    ----  	     ----  
MedianAge   	   0.669	  0.3392	  0.0962	    1.95	  (0.85;4.48)
Etiology    	  -1.476	  0.4243	  0.0132	    0.23	  (0.08;0.65)
prevalence  	  -7.362	  3.1775	  0.0597	    0.00	  (0.00;1.51)

-----------------------------------------------------------------------------------------

7 step
-----------------------------------------------------------------------------------------
Meta-Regression(Inverse Variance weights) 

 Var		  Coeff.	Std. Err.	p - value	    RDOR 	    [95%CI]
-----------------------------------------------------------------------------------------
Cte.         	   3.065	  0.5885	  0.0012	    ----  	     ----  
S            	  -0.041	  0.1572	  0.8012	    ----  	     ----  
Etiology    	  -0.796	  0.2472	  0.0146	    0.45	  (0.25;0.81)
prevalence  	  -2.831	  2.1941	  0.2379	    0.06	  (0.00;10.56)

-----------------------------------------------------------------------------------------

8 step
-----------------------------------------------------------------------------------------
Meta-Regression(Inverse Variance weights) 

 Var		  Coeff.	Std. Err.	p - value	    RDOR 	    [95%CI]
-----------------------------------------------------------------------------------------
Cte.         	   2.431	  0.3233	  0.0001	    ----  	     ----  
S            	  -0.072	  0.1554	  0.6561	    ----  	     ----  
Etiology    	  -0.742	  0.2436	  0.0159	    0.48	  (0.27;0.84)

-----------------------------------------------------------------------------------------

9 step
-----------------------------------------------------------------------------------------
Meta-Regression(Inverse Variance weights) 

 Var		  Coeff.	Std. Err.	p - value	    RDOR 	    [95%CI]
-----------------------------------------------------------------------------------------
Cte.         	   1.984	  0.7336	  0.0269	    ----  	     ----  
S            	   0.160	  0.2043	  0.4556	    ----  	     ----  
prevalence  	  -2.072	  3.7538	  0.5960	    0.13	  (0.00;723.27)

-----------------------------------------------------------------------------------------
