# Supplementary material for: The Effectiveness of Noninvasive Biomarkers to Predict Hepatitis B-Related Significant Fibrosis and Cirrhosis: A Systematic Review and Meta-Analysis of Diagnostic Test Accuracy
Source: PLoS One. 2014 Jun 25;9(6):e100182. doi: 10.1371/journal.pone.0100182 (PMC4070977; doi:10.1371/journal.pone.0100182)
Supplement: Text S7 — Meta-regression of FibroTest detecting cirrhosis. (RTF) [file pone.0100182.s016.rtf]

Text S5  Meta-regression analysis of the FobroTest for detecting cirrhosis


1 sep
-----------------------------------------------------------------------------------------
Meta-Regression(Inverse Variance weights) 

 Var		  Coeff.	Std. Err.	p - value	    RDOR 	    [95%CI]
-----------------------------------------------------------------------------------------
Cte.         	   2.932	  4.4560	  0.6295	    ----  	     ----  
S            	  -0.363	  0.3164	  0.4565	    ----  	     ----  
samplesize	  -2.690	  1.7936	  0.3744	    0.07	  (0.00;536022668.86)
MedianAge  	   2.516	  1.8368	  0.4014	   12.38	  (0.00;169377980798.77)
males        	  -0.928	  0.8312	  0.4651	    0.40	  (0.00;15268.20)
Etiology    	   1.118	  1.8079	  0.6475	    3.06	  (0.00;28957228781.37)
LBSystem    	   0.829	  0.6576	  0.4268	    2.29	  (0.00;9749.96)
LBLength    	  -0.184	  1.0059	  0.8849	    0.83	  (0.00;295665.30)

-----------------------------------------------------------------------------------------

2 step
-----------------------------------------------------------------------------------------
Meta-Regression(Inverse Variance weights) 

 Var		  Coeff.	Std. Err.	p - value	    RDOR 	    [95%CI]
-----------------------------------------------------------------------------------------
Cte.         	   2.208	  1.9330	  0.3716	    ----  	     ----  
S            	  -0.319	  0.2445	  0.3217	    ----  	     ----  
samplesize   	  -2.233	  1.0620	  0.1703	    0.11	  (0.00;10.35)
MedianAge	   2.197	  0.9486	  0.1466	    9.00	  (0.15;532.93)
males    	  -0.759	  0.6194	  0.3449	    0.47	  (0.03;6.72)
Etiology    	   1.021	  1.4172	  0.5461	    2.78	  (0.01;1234.90)
LBSystem    	   0.755	  0.5544	  0.3065	    2.13	  (0.20;23.11)

-----------------------------------------------------------------------------------------

3 step
-----------------------------------------------------------------------------------------
Meta-Regression(Inverse Variance weights) 

 Var		  Coeff.	Std. Err.	p - value	    RDOR 	    [95%CI]
-----------------------------------------------------------------------------------------
Cte.         	   2.352	  1.7908	  0.2805	    ----  	     ----  
S            	  -0.193	  0.1902	  0.3844	    ----  	     ----  
samplesize  	  -1.567	  0.5983	  0.0791	    0.21	  (0.03;1.40)
MedianAge   	   2.047	  0.8506	  0.0953	    7.74	  (0.52;116.04)
males    	  -0.374	  0.3013	  0.3031	    0.69	  (0.26;1.80)
LBSystem    	   0.493	  0.4010	  0.3064	    1.64	  (0.46;5.87)

-----------------------------------------------------------------------------------------

4 step
-----------------------------------------------------------------------------------------
Meta-Regression(Inverse Variance weights) 

 Var		  Coeff.	Std. Err.	p - value	    RDOR 	    [95%CI]
-----------------------------------------------------------------------------------------
Cte.         	   3.640	  1.4528	  0.0664	    ----  	     ----  
S            	  -0.168	  0.1891	  0.4245	    ----  	     ----  
samplesize 	  -1.755	  0.5783	  0.0386	    0.17	  (0.03;0.86)
MedianAge	   2.086	  0.8500	  0.0701	    8.06	  (0.76;85.31)
males    	  -0.480	  0.2887	  0.1718	    0.62	  (0.28;1.38)

-----------------------------------------------------------------------------------------

5 step
-----------------------------------------------------------------------------------------
Meta-Regression(Inverse Variance weights) 

 Var		  Coeff.	Std. Err.	p - value	    RDOR 	    [95%CI]
-----------------------------------------------------------------------------------------
Cte.         	   2.325	  1.2448	  0.1207	    ----  	     ----  
S            	  -0.110	  0.1931	  0.5926	    ----  	     ----  
samplesize 	  -1.750	  0.6276	  0.0385	    0.17	  (0.03;0.87)
MedianAge	   2.367	  0.8814	  0.0436	   10.66	  (1.11;102.75)

-----------------------------------------------------------------------------------------

6 step
-----------------------------------------------------------------------------------------
Meta-Regression(Inverse Variance weights) 

 Var		  Coeff.	Std. Err.	p - value	    RDOR 	    [95%CI]
-----------------------------------------------------------------------------------------
Cte.         	   0.470	  3.9971	  0.9120	    ----  	     ----  
S            	  -0.263	  0.2600	  0.3687	    ----  	     ----  
Design        	  -0.219	  0.9807	  0.8344	    0.80	  (0.05;12.23)
QUADAS      	   0.294	  0.3231	  0.4149	    1.34	  (0.55;3.29)
Prevalence	  -2.576	  2.2741	  0.3206	    0.08	  (0.00;42.01)

-----------------------------------------------------------------------------------------

7 step
-----------------------------------------------------------------------------------------
Meta-Regression(Inverse Variance weights) 

 Var		  Coeff.	Std. Err.	p - value	    RDOR 	    [95%CI]
-----------------------------------------------------------------------------------------
Cte.         	   0.605	  3.3735	  0.8648	    ----  	     ----  
S            	  -0.218	  0.2319	  0.3901	    ----  	     ----  
QUADAS      	   0.260	  0.2734	  0.3857	    1.30	  (0.64;2.62)
Prevalence	  -2.645	  1.8707	  0.2165	    0.07	  (0.00;8.70)

-----------------------------------------------------------------------------------------

8 step
-----------------------------------------------------------------------------------------
Meta-Regression(Inverse Variance weights) 

 Var		  Coeff.	Std. Err.	p - value	    RDOR 	    [95%CI]
-----------------------------------------------------------------------------------------
Cte.         	   3.774	  0.5963	  0.0007	    ----  	     ----  
S            	  -0.166	  0.2202	  0.4803	    ----  	     ----  
Prevalence	  -2.116	  1.6827	  0.2553	    0.12	  (0.00;7.40)

-----------------------------------------------------------------------------------------

9 step
-----------------------------------------------------------------------------------------
Meta-Regression(Inverse Variance weights) 

 Var		  Coeff.	Std. Err.	p - value	    RDOR 	    [95%CI]
-----------------------------------------------------------------------------------------
Cte.         	   1.083	  3.6796	  0.7784	    ----  	     ----  
S            	  -0.068	  0.2071	  0.7537	    ----  	     ----  
QUADAS      	   0.160	  0.2876	  0.5976	    1.17	  (0.58;2.37)

-----------------------------------------------------------------------------------------
 
